# Graph neural network-based breast cancer diagnosis using ultrasound images with optimized graph construction integrating the medically significant features

**DOI:** 10.1007/s00432-023-05464-w

**Published:** 2023-11-20

**Authors:** Sadia Sultana Chowa, Sami Azam, Sidratul Montaha, Israt Jahan Payel, Md Rahad Islam Bhuiyan, Md. Zahid Hasan, Mirjam Jonkman

**Affiliations:** 1 Faculty of Science and Technology, Charles Darwin University, Casuarina, NT 0909 Australia; 2 Health Informatics Research Laboratory (HIRL), Department of Computer Science and Engineering, Daffodil International University, Dhaka, 1216 Bangladesh

**Keywords:** GNN, Graph, Feature extraction, Clustering analysis, Spearman correlation, Threshold

## Abstract

**Purpose:**

An automated computerized approach can aid radiologists in the early diagnosis of breast cancer. In this study, a novel method is proposed for classifying breast tumors into benign and malignant, based on the ultrasound images through a Graph Neural Network (GNN) model utilizing clinically significant features.

**Method:**

Ten informative features are extracted from the region of interest (ROI), based on the radiologists’ diagnosis markers. The significance of the features is evaluated using density plot and *T* test statistical analysis method. A feature table is generated where each row represents individual image, considered as node, and the edges between the nodes are denoted by calculating the Spearman correlation coefficient. A graph dataset is generated and fed into the GNN model. The model is configured through ablation study and Bayesian optimization. The optimized model is then evaluated with different correlation thresholds for getting the highest performance with a shallow graph. The performance consistency is validated with k-fold cross validation. The impact of utilizing ROIs and handcrafted features for breast tumor classification is evaluated by comparing the model’s performance with Histogram of Oriented Gradients (HOG) descriptor features from the entire ultrasound image. Lastly, a clustering-based analysis is performed to generate a new filtered graph, considering weak and strong relationships of the nodes, based on the similarities.

**Results:**

The results indicate that with a threshold value of 0.95, the GNN model achieves the highest test accuracy of 99.48%, precision and recall of 100%, and F1 score of 99.28%, reducing the number of edges by 85.5%. The GNN model’s performance is 86.91%, considering no threshold value for the graph generated from HOG descriptor features. Different threshold values for the Spearman’s correlation score are experimented with and the performance is compared. No significant differences are observed between the previous graph and the filtered graph.

**Conclusion:**

The proposed approach might aid the radiologists in effective diagnosing and learning tumor pattern of breast cancer.

## Introduction

Breast cancer, a major worldwide health concern, ranks as the second leading cause of cancer-related fatalities among women (Brunetti et al. 2023; Gedik 2016). In women, it accounts for approximately 23% of all cancer types (Sayed et al. 2016). Early detection and effective treatment can greatly improve the survival rate of female patients with breast cancer (Kriti et al. 2020). Biopsy is currently the gold standard for determining whether a tumor is benign or malignant, however it is invasive (Liu et al. 2010). Different imaging modalities are used for breast cancer diagnosis at an early stage as early diagnosis increases the chances of recovery and survival (Liu et al. 2020). Because of its painless and convenient operation, absence of radiation exposure as well as effective real-time performance, ultrasonography has become a highly utilized modality in the clinical screening and diagnosis of breast cancer (Chen et al. 2023). However, because of their high sensitivity, ultrasonic instruments are susceptible to the effects of various tissues and the environment. This results in a significant amount of speckle noise, which can affect the accuracy of medical diagnosis. The likelihood of misdiagnoses ranges from 10 to 30% (Zhuang et al. 2021). Moreover, the increased number of cases causes a burden for radiologists which might contribute to incorrect interpretation and treatment. In this regard, an automated computer aided diagnosis (CAD) system may assist clinical specialists in diagnosing breast cancer effectively with less effort. Clinically significant features which radiologists analyze to diagnose breast cancer should be considered for developing a reliable automated method. An automated diagnosis based on the clinical markers that can be more effective and precise. Graph-based models have shown promising outcomes in several computer vision applications where nodes and relationships among nodes are incorporated. In this study, a novel approach is presented, proposing a graph-based model graph neural network (GNN) for categorizing breast tumors from ultrasound images into benign and malignant. This strategy can result in improved performance as along with the significant features, the relationship among them is also incorporated and the model is trained accordingly (Aswiga et al. 2021). Ten informative features are extracted from the region of interest (ROI) of breast ultrasound images: circularity (Daoud et al. 2020; Sellami et al. 2015; Ahila et al. 2022), solidity (Daoud et al. 2020; Ahila et al. 2022), Shannon entropy, GLCM (Gray-Level Co-occurrence Matrix) entropy (Berbar 2018), correlation (Ahila et al. 2022; Huang et al. 2020), dissimilarity (Ahila et al. 2022; Huang et al. 2020), contrast (Berbar 2018; Huang et al. 2020), energy (Berbar 2018; Huang et al. 2020), eclipse ratio (Daoud et al. 2020), and brightness (Sellami et al. 2015). A T test is conducted to evaluate the significance and potential relevance or discriminatory power of the features for the classification task. In addition, a density plot-based analysis is presented to evaluate the feature pattern for each class. A feature table is generated, where each row represents a single image and the columns represent the ten features. From the feature table, a graph is generated where the nodes are denoted by the rows of the feature table. The relationship among the nodes is calculated using the Spearman correlation coefficient method and the coefficient score is denoted as graph edge. Utilizing the nodes and edges, a graph is generated and fed into the GNN model for the classification. The proposed GNN model is implemented using Keras platform and optimized performing an ablation study where nine hyperparameters are tuned including hidden layer, learning rate, batch size, dropout rate, activation function, optimizer, combination type, convolutional layers and number of epochs. Bayesian optimization is further assessed for an automatic hyperparameter tuning considering quantitative parameters such as hidden layer, learning rate, batch size and dropout rate. The ablation study and Bayesian optimization results the final optimized GNN model. The optimized GNN model is further evaluated with different correlation thresholds, 0.7, 0.8, 0.95, 0.99 and 1.00, to investigate whether a higher accuracy can be achieved with a lower number of edges or a shallower graph. K-fold cross-validation is applied to assess the model’s performance consistency and the occurrence of overfitting issues. The significance of employing ROIs and handcrafted features based on the radiologist’s markers in the context of breast tumor classification is assessed through a comparative analysis. This comparison involves the evaluation of proposed model’s performance, considering handcrafted features extracted from tumor region specific ROIs and Histogram of Oriented Gradients (HOG) descriptor features extracted from the entire breast ultrasound image without selecting the region of prior tumor, as done in (Shia et al. 2021). A comparison is employed between the proposed study and the prior studies. Further experiments are conducted to assess the robustness of the model by eliminating the edges having weak connections among the similar classes and strong connections among the distinct classes. These connections might be regarded as edge anomalies, as they can have a detrimental effect on the model’s performance. A Spearman correlation score of less than 0.4 is considered as weak connection and a Spearman’s correlation score equal or greater than 0.7 is considered a strong connection. After filtering the edges based on these values, the initial graph and the filtered graph are compared with different threshold values for the Spearman correlation score. The performance of the model using the two graphs is compared to evaluate how the model performs with a filtered graph and a graph having edge anomalies. The major contributions of this work can be summarized as follows:

- A novel method to classify breast tumors using GNN model, optimized through ablation study and Bayesian optimization, with clinically significant handcrafted features extracted from the ROI is proposed.
- Feature importance analysis employing density plotting and *T* test is done.
- A graph is generated, considering each image as node and the relationship between images as edges.
- The performance of the model is improved through several thresholding experiments according to the Spearman correlation score.
- Edge anomalies based on the weak and strong connections of the same classes and distinct classes respectively are identified and filtered out.
- The robustness of the proposed approach is assessed experimenting the GNN model with two graphs: (i) graph without edge anomalies and (ii) graph with edge anomalies.

## Literature review

A number of studies have been conducted to classify breast cancer using ultrasound images through an automated approach. Singh et al. (2020) proposed the use of deep convolutional neural networks (DCNNs) for the classification of breast tumors using ultrasound images. The authors used transfer learning models to extract deep features. In order to improve the quality of the images, a despeckling preprocessing step was incorporated. The results of the study showed that the fine-tuned Inception-v3 model achieved the highest accuracy of 92.5%. Zhou et al. (2013) developed a new technique, based on the texture feature descriptors and the Shearlet transform, to increase the accuracy of breast tumor detection in ultrasound images. Liu et al. (2020) introduced an algorithm for extracting features from breast ultrasound images, which combines edge-based features and morphological feature information. The results demonstrated that the proposed algorithm outperformed traditional morphological feature methods in terms of classification accuracy (82.71%). Yu et al. (2021) presented a method analyzing the diagnostic contribution of various discriminative regions in the image. The study found that fusing deep features from different regions with the original image features significantly improved the accuracy of diagnosis from 80.8 to 85%. Aswiga et al. (2021) proposed a two-level framework for breast cancer classification based on the transfer learning techniques. The study utilized knowledge gained from nonmedical and mammography datasets as well. The proposed framework achieved an area under the receiver operating characteristic (ROC) curve of 0.89. Sellami et al. (2015) focused on extracting Breast Imaging Reporting and Data System (BI-RADS) features from a sequence of ultrasound images for the characterization of breast lesions. The results showed that the shape of the lesion changed depending on the slice, and that there were variations in the values of morphological and textural features. Moon et al. (2011) presented a computer-aided diagnostic (CAD) system for classifying breast masses in automated whole breast ultrasound (ABUS) images. The system used 3D automatic segmentation to extract texture and morphological features and achieved an accuracy of 85%. It was found that combining ellipsoid fitting features and shape features provided the best performance, with an AUC of 0.9466. Liu et al. (2010) proposed a fully automated classification method for breast ultrasound images involving two steps: ROI generation and ROI classification, using a supervised texture classification approach. The paper presented experimental results of the proposed method using a cross-validation approach and the proposed method achieved a high accuracy in classifying breast tumors. Daoud et al. (2020) proposed a method to classify breast ultrasound images using a combination of deep features extracted from a pretrained CNN model and conventional handcrafted features. The study indicated that the best combination of deep features is obtained with a feature set that includes convolution features extracted from all convolution blocks of the VGG19 model. In the study of Shia et al. (2021), a machine learning method was proposed for the classification of benign and malignant breast tumors in ultrasound images without requiring a priori tumor region selection. The proposed method had a high classification performance, achieving a sensitivity of 81.6%. Telagarapu and Poonguzhali (2018) proposed an algorithm for detecting breast cancer in ultrasound images, utilizing filtering and feature extraction techniques. The results showed that the support vector machine (SVM) classifier outperformed the Fuzzy K-nearest neighbor (KNN) classifier with an accuracy of 87.3%, for the texture features extracted using the Tetrolet transform. Shia and Chen (2021) proposed a transfer learning method for classifying benign and malignant breast tumors using breast ultrasound images. The method involved a pretrained deep residual network model for image feature extraction and a linear SVM model for classification. The proposed approach achieved a sensitivity of 94.3%. Zhuang et al. (2021) presented a breast ultrasound classification using image decomposition and fusion techniques with adaptive spatial feature fusion technology. The proposed method had the best performance, with an accuracy of 95.48%. Cao et al. (2019) evaluated the effectiveness of different deep learning architectures for the classification of breast lesions in ultrasound images. For the classification task, DenseNet was identified as the most suitable deep learning architecture. Ahila et al. (2022) presented a CAD system, based on a wavelet neural network and the grey wolf optimization algorithm, to detect abnormalities in breast ultrasound images. The proposed system achieved a high classification accuracy, of 97.4%, in distinguishing malignant and benign breast lesions using ultrasound images. Mohammed et al. (2018) proposed a computerized system for breast cancer characterization using multifractal dimensions and back propagation neural networks on ultrasound images. The proposed system achieved a precision of 82%, a sensitivity of 79.3%, and a specificity of 84.7%. Table 1 shows the dataset, method, result, contribution, and limitation of the prior works.

**Table 1:** Literature review

Author	Dataset	Method	Results obtained	Contribution	limitation
Kriti et al. (2020)	Not mentioned	DCNNs	Accuracy: 92.5%	Utilizing DCNNs for Breast Tumor Classification and Transfer Learning for Feature Extraction	Limited diversity of data Pretrained CNNs was not used for the comparison analysis with the proposed model
Liu et al. (2020)	Ultrasound Diagnostic Instrument (71 malignant tumors and 121 benign tumors)	(Classification with support vector machine (SVM) using morphological feature and edge-based features)	82.69% with edge-based features	Proposed algorithm's effective in identifying and classifying breast tumors	No feature testing was performed
Yu et al. (2021)	479 breast ultrasound images (356 benign and 123 malignant)	Pretrained Inception-V3 network	Accuracy: 85% AUC:87.2%	Three discriminative regions were identified and analyzed	The marginal zone and the posterior echo region of the breast ultrasound image were not considered Did not explore multicenter datasets
Aswiga et al. (2021)	MIAS and BCDR dataset	Multilevel transfer learning (MLTL) Feature extraction-based transfer learning (FETL) GLCM-based feature extraction	ROC curve: 0.89	Proposed two-level framework for breast cancer classification	Canny edge detection and GLCM-based feature extraction techniques may not capture all the relevant information Analysis of potential impact of training and testing data on the classification performance was not provided
Daoud et al. (2020)	Training: 380 images Testing: 163 images	Two-phase procedure- Deep features were extracted from the pretrained VGG19 model at six different extraction levels and extracted deep features processing using feature selection	Accuracy: 96.1% Sensitivity: 95.7% Specificity: 96.3%	Combining deep features with conventional handcrafted features, texture features, and morphological features	Used a specific pre-trained model (VGG19) for extracting deep features No performance comparison with other transfer learning models were addressed
Shia et al. (2021)	677 ultrasound images (312 images benign and 365 malignant)	Computer‑Aided Diagnosis (CAD)	Sensitivity: 81.64% Specificity: 87.76% Positive Predictive Value: 84.1% Negative Predictive Value: 85.8%	Eliminate the need for manual selection of tumor regions	Not mentioned the classification accuracy Not selected the features based on the radiologist markers
Shia and Chen (2021)	543 patients (benign 302 and malignant 241)	Pretrained Deep Residual Network for feature extraction linear SVM model for classification	Sensitivity: 94.3% Specificity: 93.22% AUC:93.8% Positive Predictive value: 92.6% Negative Predictive Value: 94.8%	Accurately classifying malignant tumors of BI-RADS category 4	Extracted malignant features were not synonymous with the BI-RADS malignant lexicons, which represents a clinical limitation Not used k-fold cross validation to test model’s overfitting issue
Zhuang et al. (2021)	1328 breast ultrasound images	Image decomposition and fusion techniques with adaptive spatial feature fusion	Accuracy: 95.48% Precision: 98.11% Specificity: 98.33% Sensitivity: 93.92% F1 score: 95.71%	Adaptive Spatial Feature Fusion outperformed other methods	Dataset did not fully represent the diversity of breast cancer cases The study did not address inherent speckle noise in breast ultrasound images The study did not provide information on false positives/false negatives
Ahila et al. (2022)	346 patients (249 malignant and 97 benign)	Wavelet Neural Network and Grey Wolf Optimization algorithm to detect abnormalities	Accuracy: 98% Sensitivity: 98.8% Specificity: 95.9% Positive Predictive Value: 98.4% Negative Predictive Value: 96.9%	Automated CAD system using a metaheuristic algorithm and machine learning Optimization of the WNN with the GWO algorithm to improve classification accuracy	Specific preprocessing was not mentioned Not mentioned the hyperparameter tuning of the proposed model
Mohammed et al. (2018)	184 breast ultrasound images (72 abnormal tumor and 112 normal)	Multifractal Dimensions and Back Propagation Neural Networks	Precision: 82.04% Sensitivity: 79.39% Specificity: 84.75%	Presented an automated characterization of breast cancer	Limited number of cases for both classes in the dataset No performance comparison is shown with the previous studies
Sahu et al. (2023)	Mini-DDSM for mammogram and BUSI for breast ultrasound images	Five deep hybrid convolutional neural network-based framework	Accuracy(mini-DDSM) Abnormality: 99.17% Malignancy: 98.00% Accuracy (BUSI) Abnormality: 96.52% Malignancy: 93.18%	Hybridized two efficient deep CNN networks	Limited diverse feature learning ability of the deep CNN model Possibility of false detection when the second classifier is employed Required the activation of at least two networks for each test image, which increase computational complexity
Zhou et al. (2022)	BP, BUSI and HCUS dataset	Lightweight Attention Encoder–Decoder Network (LAEDNet)	Dice Coefficient Score (DSC) (BP): 73.0% DSC BUSI): 73.8% DSC (HCUS): 91.3%	Designed lightweight decoder block, LRSE, with the guidance of attention scheme	Model lightweight architecture prioritize implementing efficiency over achieving the highest possible segmentation accuracy hyperparameters tuning was not mentioned
Sirjani et al. (2023)	Three public datasets—BUSI, BUS and Public dataset of 86 breast cancer ultrasound images	InceptionV3 network	Precision: 83.0% Recall: 77.0% F1 score: 80.0% Accuracy: 81.0% AUC: 81.0% Root Mean Squared Error: 18.0% Cronbach’s α: 77.0%	Converted InceptionV3 modules to residual inception ones	The study did not provide detailed information on the size and diversity of the datasets used No comparison with other Deep learning models
Atrey et al. (2023)	86 images (43 mammogram and 43 ultrasound images.)	Cubic Support Vector Machine (SVM)	Accuracy: 98.84% for combined dataset of mammogram and ultrasound Mammogram’s accuracy: 93.41% Ultrasound’s accuracy: 91.67%	Redundant features were determined from forty-two grayscale extracted texture features	Manual extraction of Region of Interest (ROI) The study did not explore image enhancement techniques to improve the classification accuracy The study did not mention any comparison of selected features with medical features

Within the domain of breast tumor classification, the proposed method introduces an intriguing and distinctive approach characterized by its notable strengths. While the literature has explored various techniques, each approach carries its own set of advantages and limitations. For instance, (Yu et al. 2021) enhanced accuracy by fusing deep features from specific regions of breast ultrasound images, although their focus was on selecting regions limited comprehensive image analysis. Similarly, (Daoud et al. 2020) integrated deep features with conventional handcrafted ones, yet considerations of feature relevance and redundancy were lacking. Meanwhile, (Shia and Chen 2021) harnessed deep learning effectively but faced challenges aligning extracted malignant features with clinical standards. Significantly, the proposed method utilizes GNN to capitalize on clinically significant features extracted from the ROI in ultrasound images. This approach exhibits strength through rigorous feature selection, statistical evaluation of feature significance, and a comprehensive understanding of image relationships via a graph-based representation. The GNN model's ability is optimized through correlation threshold tuning, results in remarkable accuracy, precision, recall, and F1 score. Unlike previous research, which frequently suffers from limitations such as restrictive feature selection, redundancy, greater computational complexity or a lack of diverse datasets, this GNN-based technique presents a viable avenue for robust breast tumor classification, with the potential to improve early diagnosis and clinical decision making.

## Materials and methods

### Dataset description

**Fig. 1:**
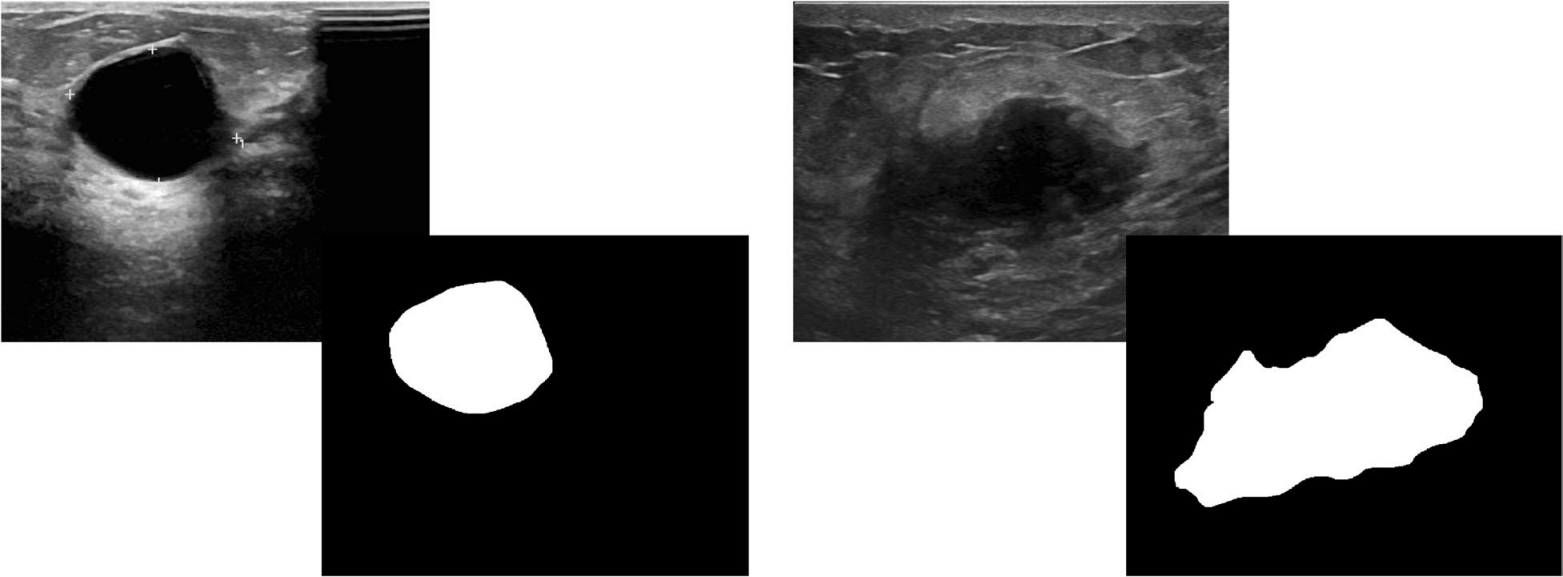
Sample image of benign class and malignant class with their corresponding ground truths

This study uses a publicly available breast ultrasound dataset of 780 PNG images (Al-Dhabyani et al. 2020). The images are of female patients with an age in the range of 25–75 years. The dataset has an average image resolution of 500 × 500 pixels and is categorized into normal, benign, and malignant classes with 266, 467, and 210 images, respectively. The ultrasound images are generated using high-end imaging instruments. The ground truth for the benign and malignant classes is provided. Only the benign and malignant classes are used in this study to analyze the tumor pattern of breast cancer, as the normal class does not contain any tumors. Therefore, in this research, 647 images are used. Sample images are shown in Fig. 1.

### Proposed methodology

**Fig. 2:**
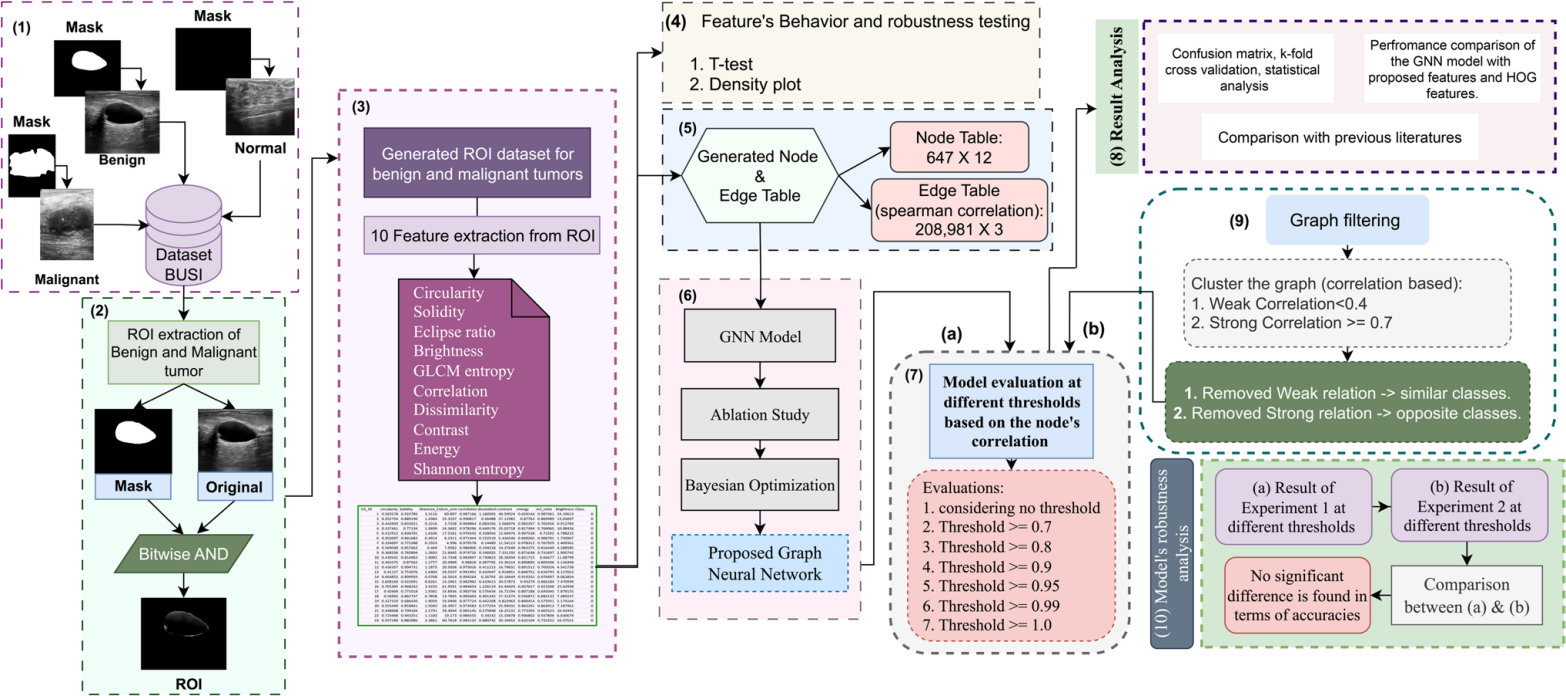
The proposed framework for the breast cancer classification

This study presents an automated approach to classify benign and malignant tumors based on a graph of clinical features, using a GNN model. An overview of the methodology is presented in Fig. 2.

In this study, a breast cancer ultrasound dataset is used and the experiments are carried out using benign and malignant classes. The ROI is extracted from the images using a bitwise AND operation between the raw images and the image ground truths. Ten informative and clinically relevant features are extracted from the ROI and a feature table is created, based on these features. A density plot is utilized to demonstrate how particular features influence distinguishing classes. The features are evaluated using the T test statistical analysis method. A graph is generated from the feature table, where the node table dimensions are 647 × 12 and edge table dimensions are 208,981 × 3. In this graph, the nodes are denoted by the images with their corresponding features. Hence, the node table has 647 rows, where each row represents a particular image. The 10 columns of the feature table represent the ten features for each image, in addition to a unique ID for each image and a class label. The edges are determined by deriving the node-to-node relationship for each node, using the Spearman correlation coefficient method. A GNN model is proposed to implement the classification task using the node and edge table. The GNN model is optimized through an ablation study, finetuning nine hyperparameters. Further, a Bayesian optimization is employed for an automatic quantitative hyperparameter tuning which validates the outcome of the ablation study and results the optimized GNN model. The performance of the optimized model is analyzed with the generated graph, using different thresholds for the Spearman correlation score to evaluated whether the number of edges can be reduced and a higher accuracy can be achieved with lower complexity. The performance of the model is analyzed using several performance metrics. To understand the importance of evaluating ROIs for the classification task, a comparison is conducted between the performance of proposed model based on the handcrafted features, extracted from tumor ROIs and HOG descriptor features extracted from full image of breast ultrasound. Comparison with the previous studies is also carried out with the proposed study. Lastly, to assess the robustness of the model, a clustering analysis is performed on the graph where the weak connections among the similar classes and strong connections among the distinct classes are removed. Correlations less than 0.4 are considered weak and correlations equal or greater than 0.7 are considered strong. After filtering out edges based on these two criteria, the first graph and the filtered graph are applied with several thresholds based on the Spearman correlation score.

### Feature extraction

In the diagnosis of breast cancer, clinically significant features play a crucial role. Benign and malignant tumors exhibit differences in several characteristics such as their structure, shape and border (He et al. 2023). In this paper, we propose a set of features, listed in Table 2, that can be used to distinguish benign and malignant masses and considered clinically relevant.

**Table 2:** Description of the features

Features		Description
Morphological features	Circularity	Geometrical property-based feature
	Solidity	Geometrical property-based feature
	Eclipse ratio	Geometrical property-based feature
	Brightness	Intensity-based feature
GLCM features	GLCM entropy	Texture-based feature
	Correlation	Texture-based feature
	Dissimilarity	Texture-based feature
	Contrast	Texture-based feature
	Energy	Texture-based feature
	Shannon entropy	Texture-based feature

### Morphological features

Morphological features describe the shape and texture of tumors and can be highly effective in differentiating between benign and malignant tumors. Malignant tumors typically exhibit irregular shapes, spiculated margins, and a heterogeneous internal texture (Meng et al. 2023), whereas benign tumors tend to have more regular shapes, smooth margins, and a homogeneous texture (Zhang et al. 2023).

### Circularity

Circularity is a measure for the degree of similarity of a shape to a perfect circle and is often used as an indication of the regularity of a tumor’s shape (Sellami et al. 2015). Malignant tumors typically have low values for circularity, whereas benign tumors have higher values for circularity (Daoud et al. 2020). Equation (1) estimates circularity by taking the tumor area (${a}_{T}$) and the major axis length (${d}_{max}$) of the equivalent tumor ellipse, which is defined by the region of interest's major and minor axis lengths (Sellami et al. 2015).

1 $$\mathrm{Circularity}= \frac{4\times {a}_{T}}{\pi \times {{d}_{max}}^{2}}.$$

### Solidity

**Fig. 3:**
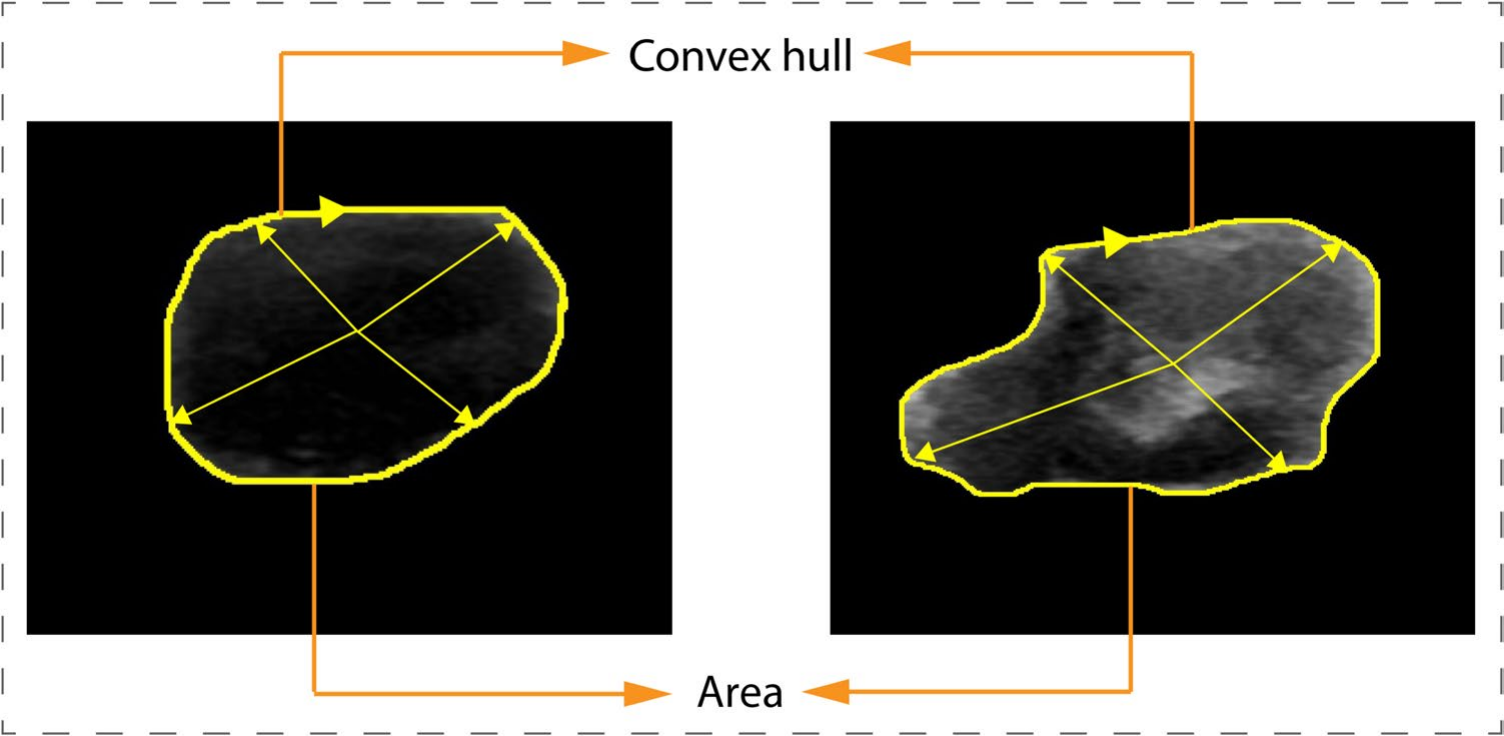
Solidity feature for Benign and Malignant tumors

Solidity is a measure of the compactness of the tumor shape and is calculated as the ratio of the object’s area to the number of pixels in its convex hull as in Eq. (2), see Fig. 3 (Triyani et al. 2023). Benign tumors generally have a higher solidity compared to malignant tumors because malignant tumors often have an irregular and branching shape, resulting in a lower solidity value (Liu et al. 2022).

2 $$\mathrm{Solidity }= \frac{\mathrm{Tumor'}\,\mathrm{s area}}{\mathrm{Convex \,hull}}.$$

### Eclipse ratio

**Fig. 4:**
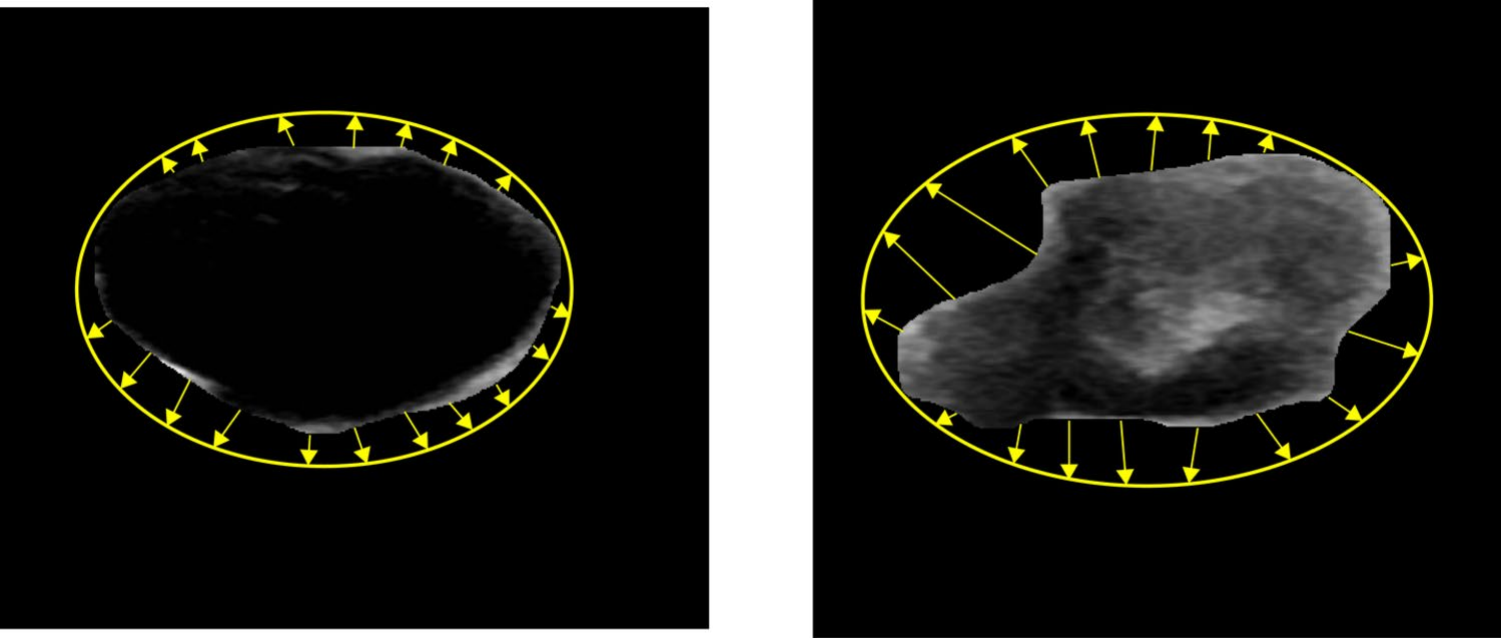
Eclipse ratio feature for Benign and Malignant tumors

The ratio of the ellipse perimeter is another morphological feature that can be used to differentiate between benign and malignant tumors. The eclipse is drawn covering the outer points of the tumor edge. This feature is calculated by considering the ratio of the area of eclipse and the area of the tumor. Because of their irregular boundaries, malignant tumors have a higher eclipse ratio than to benign tumors (Daoud et al. 2020). Figure 4 illustrates the differences in area between the eclipse and tumor.

### Brightness

Brightness is an intensity-based feature affected by the contrast between different regions in an image, which can affect the measurement of texture features. In breast lesion images, the boundary can either have an abrupt interface or an echogenic halo. An abrupt interface is marked by a distinct echogenic line, while an echogenic halo does not show a clear boundary between the lesion and the surrounding tissue (Boulenger et al. 2023). To evaluate the sharpness of the boundary or brightness, a distance map can be used to distinguish the outer part of the lesion from the surrounding tissue. Equation (5) defines the brightness with the number of grey level pixels, g(*p*) in the surrounding tissue ${S}_{\mathrm{tissue}}$ and the number of pixels in the outer part of the lesion ${N}_{\mathrm{out}}$ (Sellami et al. 2015).

3 $${\mathrm{avg}}_{\mathrm{tissue}}= \frac{{\sum }_{\mathrm{distance}\left(p\right)=1}^{k}g(p)}{{S}_{\mathrm{tissue}}},$$

4 $${\mathrm{avg}}_{\mathrm{out}}= \frac{{\sum }_{\mathrm{distance}\left(p\right)=1}^{k}g(p)}{{N}_{\mathrm{out}}},$$

5 $$\mathrm{Brightness}= \left|{\mathrm{avg}}_{\mathrm{tissue}}- {\mathrm{avg}}_{\mathrm{out}}\right|.$$

### GLCM features

**Fig. 5:**
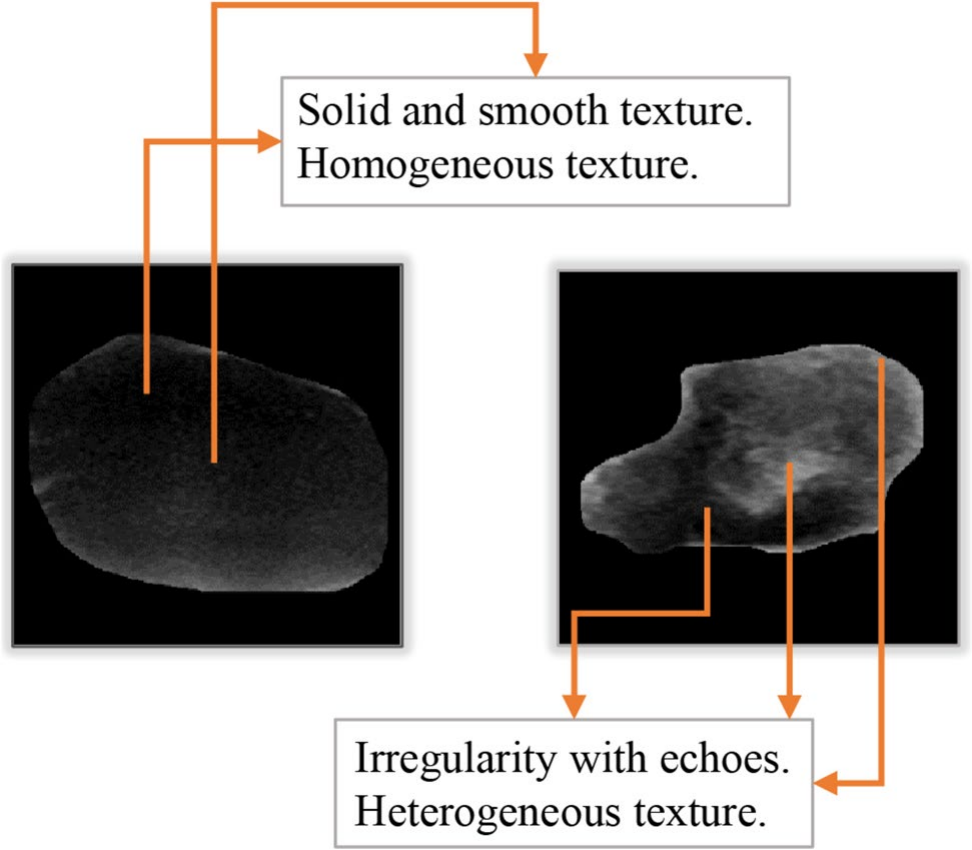
Visualization the textures of benign and malignant tumor

The GLCM features capture the texture patterns and spatial relationships between pixels. Benign and malignant tumors can be visually categorized by analyzing the lesion, texture, intensity, and complexity of the tumors (Mendelson et al. n.d.). Malignant tumors typically exhibit a higher distribution of black and grey pixels, whereas benign tumors tend to be more solid and exhibit a regular shape with a dense visualization of the tumor. In Fig. 5, we can see that the malignant tumor has highly irregular boundaries, a heterogeneous internal texture, and internal echoes, compared to benign tumor, see Fig. 5.

### Shannon entropy

Shannon entropy is a measure of uncertainty in the texture of an image, which can reflect the complexity and presence of information of a tumor (Rafid et al. 2022). Malignant tumors usually have more complex and irregular textures, resulting in higher Shannon entropy values than benign tumors.

### GLCM entropy

The GLCM is a technique used in texture analysis to quantify the relationship between two pixels with gray levels *x* and *y* that are separated by a distance D in direction *z* within an image. The gray-level co-occurrence matrix records the number of times the pair of gray levels (*x*, *y*) appears at a distance D apart in the subimage, with the result being stored in the entry (*x*, *y*) of the matrix. To scale the values in the image into G levels, they are converted into integers between 1 and *G*. The size of the gray-level co-occurrence matrix, which is *G* $\times$ *G*, is determined by the *G* numbers of gray levels. GLCM is applied with varying directions *z* {0, *π*/4, *π*/2, 3*π*/4} and distance D, resulting in a set number of D GLCMs each of size *G* $\times$ *G* for each given *z* (Berbar 2018).

### Correlation

**Fig. 6:**
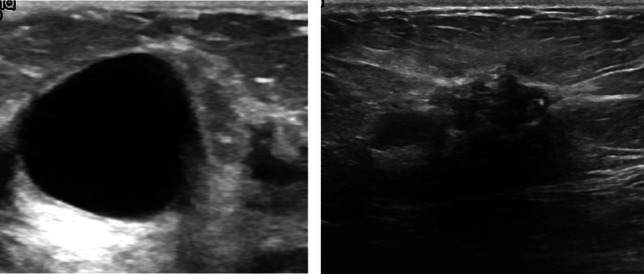
The tumors correlation with the surroundings. **a** Benign; **b** Malignant

In the context of breast cancer detection, correlation is used to assess the similarity of pixel values between the tumor and the surrounding tissue. By analyzing the GLCM, correlation in Eq. (6) can be used to distinguish between benign and malignant lesions (Berbar 2018). Malignant tumors typically exhibit a higher degree of correlation (Fig. 6) between pixels, while benign tumors have lower correlation values.

6 $${C}_{or}= \sum\limits_{i,j=0}^{l-1}{r}_{ij}\left(i-{m}_{g}\right)\left(j-{m}_{g}\right).$$

### Dissimilarity

Dissimilarity measures the degree of dissimilarity between two images or regions of interest. It can be used to identify differences in tissue structures. These differences may indicate irregularities in shape or texture, which can be indicative of cancerous growths. Dissimilarity, see Eq. (7), is based on the GLCM, which is a second-order histogram used to describe image textures. The matrix can be represented as an asymmetric matrix that quantifies the pairwise distribution of pixels (Berbar 2018).

7 $$D= \sum\limits_{i,j=0}^{l-1}{r}_{ij}\left|i-j\right|.$$

### Contrast

Tumors or abnormal growths often exhibit different contrast values compared to healthy tissue, making contrast a useful tool in identifying anomalies (Berbar 2018). Ultrasound images can also benefit from contrast features for the differentiation, as malignant lesions tend to have a more varied echogenicity and irregular shape compared to benign lesions. The contrast feature in Eq. (8) quantifies the joint distribution of the pairwise pixels in an image (Huang et al. 2020).

8 $${C}_{o}= \sum\limits_{i,j=0}^{l-1}{r}_{ij}{(i-j)}^{2}.$$

### Energy

Energy is a measure of the uniformity in the pixel intensities of an image. Ultrasound imaging measures the backscatter of sound waves from tissue. The amount of backscatter is related to the tissue's acoustic properties, including its density, composition, and structure (Berbar 2018). Malignant tissues often have a more disordered structure than benign tissues, leading to increased backscatter and higher energy levels in the ultrasound image. Energy (see Eq. 10) is based on the gray histogram that explains how the image's gray scale is distributed. In Eq. (9), $q(i)$ denotes the number of pixels in the ith grayscale, n denotes the total number of pixels, and l denotes the gray level (Huang et al. 2020).

9 $$r\left(i\right)= \frac{q(i)}{n}, \quad i=0, 1, \dots , l-1,$$

10 $$E= \sum\limits_{i=0}^{l-1}{[r\left(i\right)]}^{2}.$$

### Feature analysis

A set of 10 informative features are extracted from the ROI of breast ultrasound image. To evaluate the significance of the features, the density plot is utilized to evaluate how the influence of particular features in distinguishing classes.

### Density plot for feature visualization

**Fig. 7:**
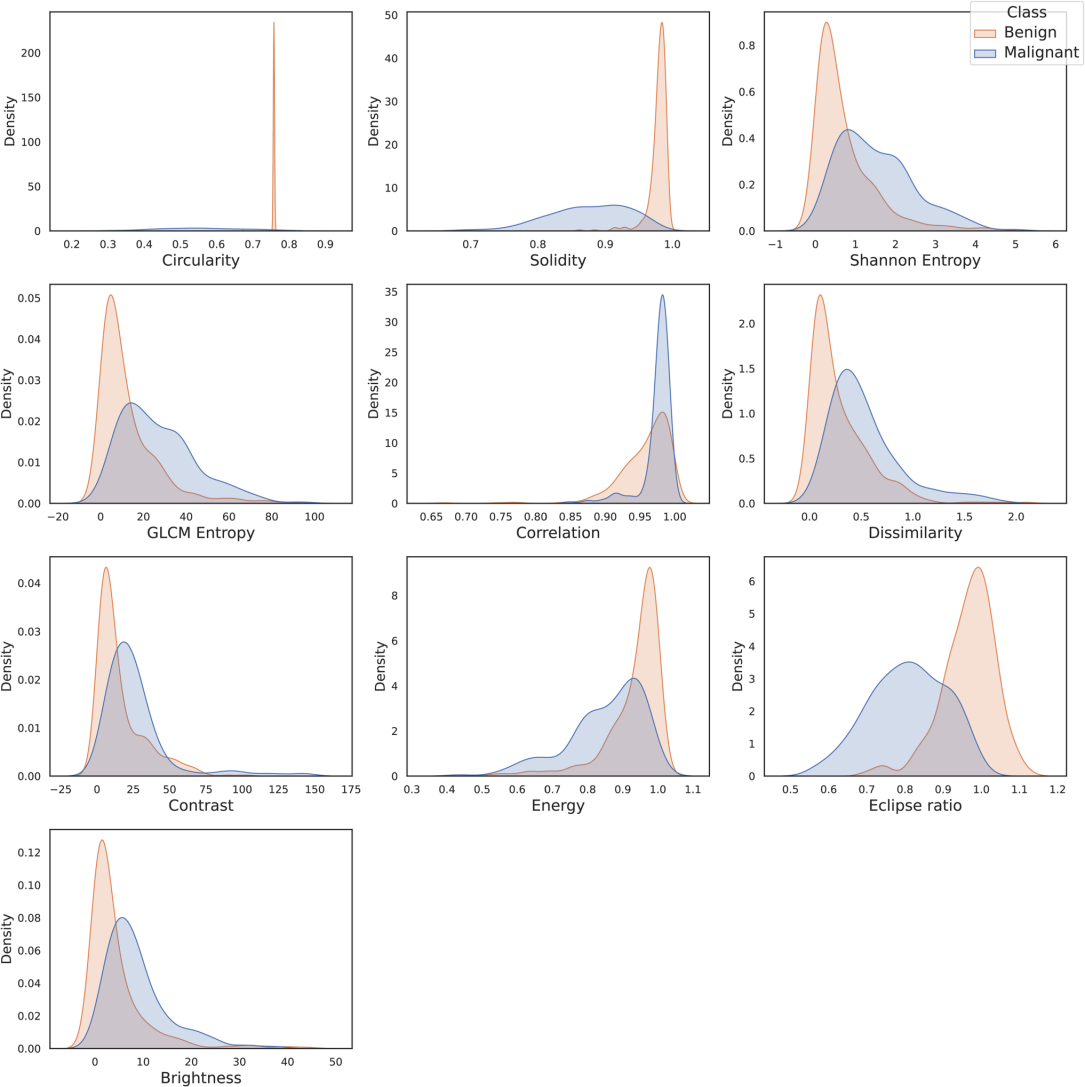
Density plots to differentiate the change in feature distribution pattens for benign and malignant tumors

In Fig. 7, the orange curve represents the feature values of class benign and the blue curve represents the feature values of class malignant. The distribution of the values for malignant tumors appears to be much wider than that of benign tumors. For Circularity, the threshold range for malignant tumors is from 0.3 to 0.81 whereas the threshold range for benign tumors is 0.75 to 0.8. Similarly for solidity, threshold range for malignant tumor is 0.68 to 0.97 and for benign 0.86 to 0.99. For Shannon entropy, the threshold range for malignant tumors is 0.03 to 4.95 and for benign 0.046 to 4.68 where most values are between 0.046 and 1.5. For correlation, values of the benign class range from 0.97 to 0.99, whereas the threshold range for classifying a tumor as malignant is 0.9 to 0.99. The rest of the features are also distinct enough to assign values to a particular class. Based on the visualization, it can be inferred that the feature values for benign tumors exhibit a consistent pattern and show regularity, whereas the malignant tumors demonstrate significant deviations from this pattern and a nonuniform distribution.

### Feature testing

The T test is a statistical analysis method which is employed in this study to evaluate the features. To assess whether there are significant differences between the two groups, the *p* values are calculated (Effrosynidis and Arampatzis 2021). Table 3 lists the *T* test results for the features.

**Table 3:** Feature robustness analysis through *T* test

Feature	*t* value	*p* value
Circularity	− 35.064039	1.467754e-151
Solidity	− 34.372150	6.399186e-148
Shannon entropy	11.420615	1.219706e-27
GLCM entropy	11.169152	1.330398e-26
Correlation	6.112874	1.694358e-09
Dissimilarity	9.664385	1.000733e-20
Contrast	6.319524	4.894419e-10
Energy	− 10.674455	1.319478e-24
Eclipse ratio	− 23.557379	5.206523e-89
Brightness	7.810727	2.311481e-14

A low *p* value indicates that the feature is a strong discriminator between the classes (Lim and Kim 2021). The *p* values associated with our feature set *f*{..} are much smaller than the threshold of 0.05 (*p*_value(*f*{..}) <  < 0.05). This indicates that this is a robust discriminative feature set to accurately classify breast cancer.

### Graph construction

To generate the graph, first a feature table is created with 647 rows and 12 columns. The 12 columns include 10 features, a unique ID for the rows and the target class. Considering each row as an individual node ($$v),$$ the node numbers of the malignant images fall within the range of 1 to 210 and the node numbers of the benign images are within the range of 211 to 647. An edge (*E*) table is constructed calculating the Spearman correlation coefficient between the rows. This quantifies the relationships between the images, based on the features of each image. The connection is derived by calculating the coefficient scores of each row for example node 1 to node 2, node 1 to node 3,… node 1 to node 647 and node 2 to node 3, node 2 to node 4,… node 2 to node 647. The Spearman correlation coefficient between two nodes is calculated each time and the connection of a particular node with all the other nodes is found. The resultant edge table consists of 208,981 rows and 3 columns. The Spearman correlation coefficient (S) is calculated as follows.

11 $$S=1- \frac{6 \sum {d}_{v}^{2}}{n \left( {n}^{2}-1 \right)}.$$

Here ${d}_{v}$ denotes the differences between the nodes and n represent the number of nodes. The correlation patterns between the nodes are outlined in the edge table, see Table 4.

**Table 4:** Edge table for the graph

Number of edges	Source	Target	Correlation value
0	1	2	0.835165
1	1	3	0.604396
2	1	4	0.862637
3	1	5	0.818681
4	1	6	0.670330
…	…	…	…
208,976	644	646	0.917582
208,977	644	647	0.434066
208,978	645	646	0.945055
208,979	645	647	0.714286
208,980	646	647	0.554945

Table 4 shows how an edge between two nodes is denoted, based on a correlation score. “Source” stands for the source node, “Target” denotes the target node, and “correlation”, the Spearman correlation value between the target and source nodes.

With the set of nodes ($${v}_{n})$$ and edges ($${e}_{m})$$, a graph *G* = (*V*, *E*) is generated where $${v}_{n}$$ ∈ *V* and $${e}_{n}$$ ∈ *E*, *n*, *m* denote the number of nodes and edges respectively. In a graph, the edges are constructed as $${e}_{ij}$$ = ($${v}_{i}, {v}_{j})$$, representing the relationship between node $${v}_{i}$$ and$${v}_{j}$$. An adjacency matrix (*A*) is also generated from the graph (*G*) with *n* × *n* dimensions. If $${A}_{ij}=1,$$ there is an edge between two nodes ($${e}_{ij}$$ ∈ *E*) and $${A}_{ij}=0$$ if $${e}_{ij}$$ ∉ E. Excluding the unique feature ids and target classes, the 10 features form the feature vectors X of the graph where $${X}_{v}$$ represents the feature vector of a particular node (*v*) (Wu et al. 2021).

**Fig. 8:**
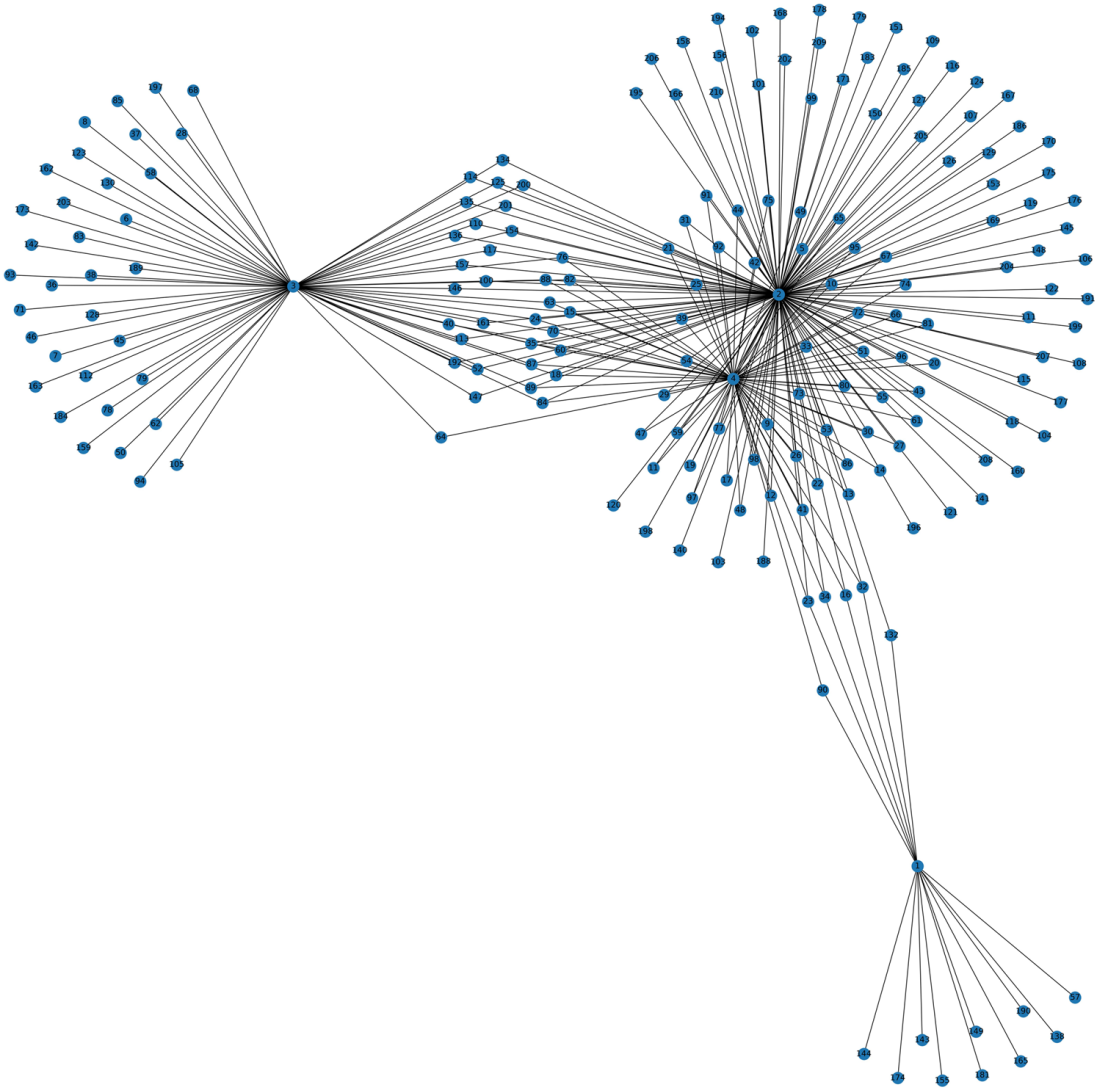
A visualization of a small portion of the graph

As the graph is generated based on the image-to-image relationships, the resultant graph is complex and large. A small portion of the graph with 198 nodes and 300 edges is shown in Fig. 8.

### Model

The proposed GNN model can be adapted to diverse domains by capturing internode connections to perform a wide range of tasks (Asif et al. 2021; Grattarola and Alippi 2021).

### Feed forward network (FFN) block

An optimized FFN is utilized for more effective learning of the node representation. It is an off-the-shelf classifier (Gayathri et al. 2022) where the layers are densely connected. This facilitates the learning of complex patterns and relationships within the data. It is a type of one directional network, consisting an input layer, hidden layers and an output layer, where the information flow starts from the input layer and is transformed through a series of hidden layers. Each layer applies a set of weights and biases to the output of the previous layer (Truong et al. 2020) and with these three types of layers a FFN block is constructed. Optimized FFN blocks help to learn complex nonlinear transformations of node representations through a GNN model, increasing the ability to capture intricate patterns in the graph data.

**Fig. 9:**
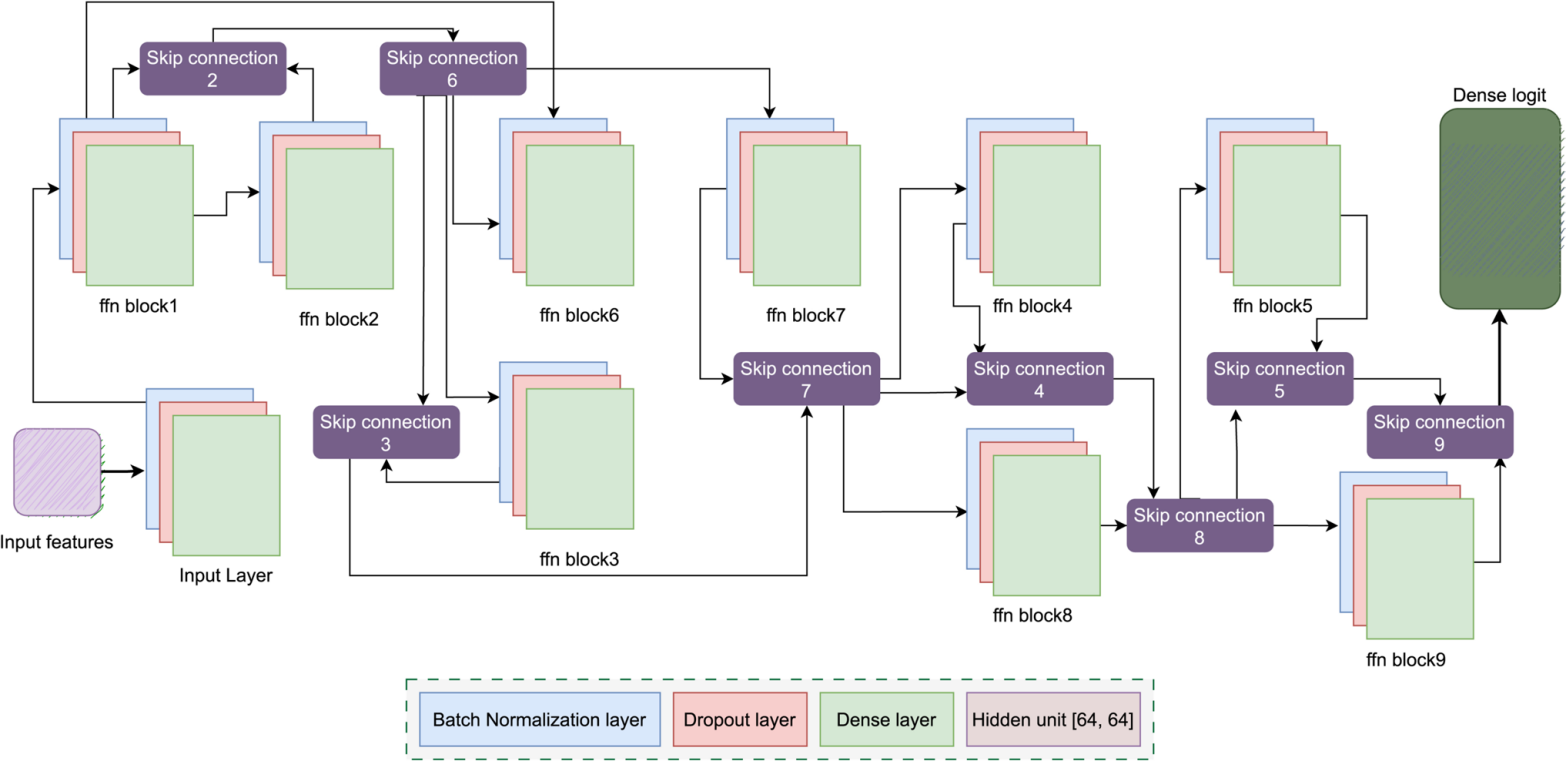
The optimized FFN block connections used for the GNN model

Our proposed FFN block consists of three fully connected hidden layers, including the batch normalization layer, a dropout layer with the dropout rate of 0.2, and a dense layer with [64, 64] dimensions and an Exponential Linear Unit (ELU) activation function. These layers form a fully connected network for each block. The proposed GNN model has nine FFN blocks, each block containing corresponding skip connections. A skip connection allows information to be passed directly from one block to another without being transformed. The input layer takes the 10 features as input feature which are then forwarded to FFN block 1. The FFN blocks work in such a way that the output of the previous FFN block is passed through a skip connection to the next FFN block. The final output is passed through a Softmax activation function to produce a probability distribution for the possible node labels. Figure 9 shows the architecture of the FFN block connections.

The Fig. 9 depicts the architecture of the FFN blocks with a connection that is optimized for this study. The FFN consists of an input layer that takes the input features and feeds them through a sequence of 9 FFN blocks, each comprising of nine skip connections and three fully connected layers.

### Proposed GNN model

The GNN model learns the representation of a graph by aggregating the feature vectors of a node's neighboring nodes by capturing the neighborhood information, which is used to generate an updated node embedding. This process is called message passing. Each node of the graph gets a unique embedding, generated from its neighborhood information. Message passing is done through the convolutional operation of the GCN layer. The equations for the message passing process are Eqs. (12), (13), and (14) (“The Graph Neural Network Model,” n.d.):

12 $${h}_{u}^{i}= \sigma \left( {W}_{n}^{i} {h}_{u}^{i-1}+ {W}_{Nei}^{i} \sum_{v\epsilon N\left(u\right)}{h}_{v}^{i-1}+ {b}^{i}\right),$$

13 $${h}_{u}^{(i+1)}= {Update}^{(i)} \left({h}_{u}^{\left(i\right)} , {AGGREGATE }^{\left(i\right)}\left(\left\{{h}_{u}^{\left(i\right)}, { \forall }_{u }\in N\left(u\right)\right\}\right)\right),$$

14 $${=Update}^{i} \left({h}_{u}^{i} , {m}_{N\left(u\right)}^{\left(i\right)}\right),$$

**Fig. 10:**
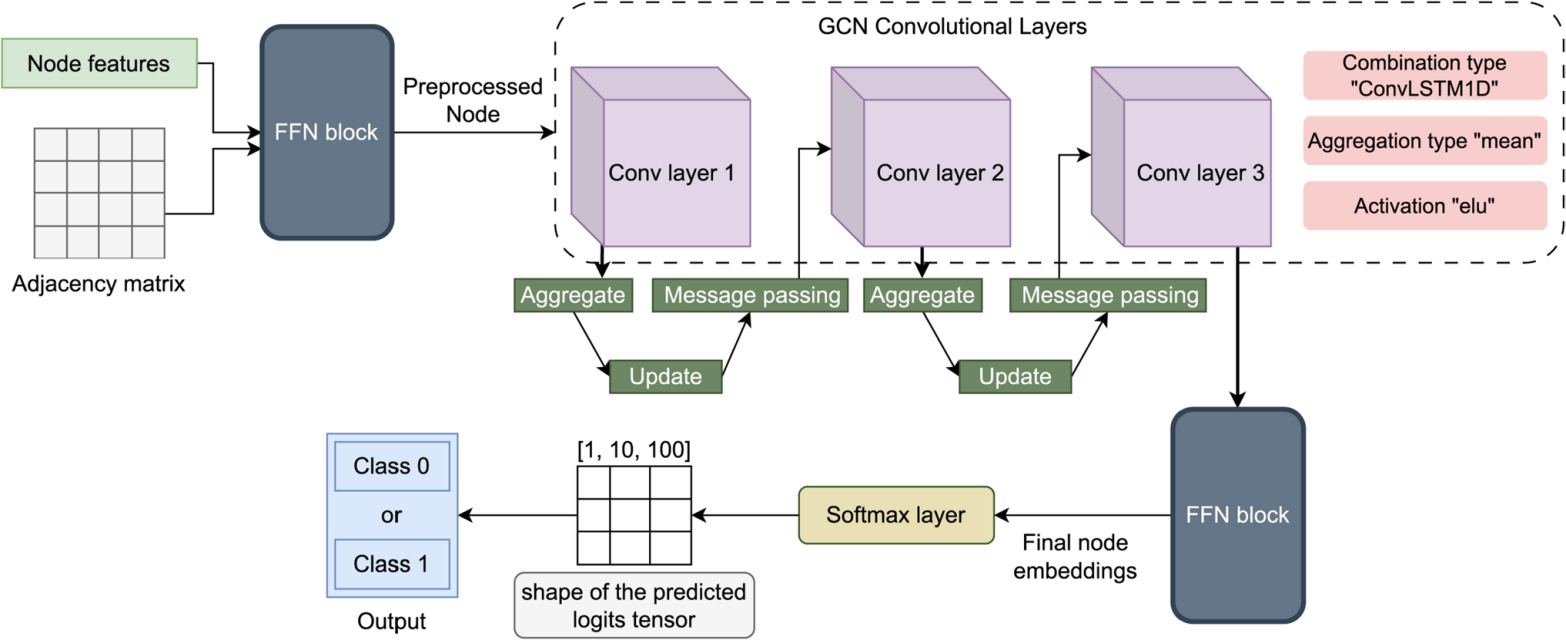
Proposed GNN model architecture

${h}_{u}^{i}$ denotes ${i}^{th}$ hidden embedding for node u. Its previous node embedding is ${h}_{u}^{i-1}$. ${W}_{n}$, ${W}_{Nei}$ and b represent the message passing parameters at iteration i while elementwise nonlinearity is represented by $\sigma$. $\sum_{v\epsilon N\left(i\right)}{h}_{v}^{i-1}$ is the embedding vector neighboring node of u ($N\left(u\right))$. Equations (12) and (13) describe the updating process the node embedding or message passing. ${m}_{N(u)}^{(i)}$ is the message that is aggregated from the neighbor N(u). The previous hidden embedding will be updated from ${h}_{u}^{(i)}$ to ${h}_{u}^{(i+1)}$ (updated embedding). The updated node embeddings are normalized to get a more informative node feature vector. After getting the final nonlinear node embeddings as output of GCN layer, they are fed into the densely connected FFN. The FFN then applies nonlinear transformations to the inputs for the final prediction task. The hidden units of the dense layers use an ELU activation function, which allows the model to learn complex, nonlinear relationships between the input features. The output of the GNN model can be represented as follows (Asif et al. 2021),

15 $$O=F\left({h}_{u}, {f}_{u}\right),$$

where O is the output or the predicted label done by the FFN. The function F(${h}_{u}, {f}_{u}$) represents the feed-forward network where ${h}_{u} and {f}_{u}$ are the node embeddings and feature vectors for each node u respectively. Figure 10 shows the proposed GNN model framework.

In the proposed GNN model, the node feature vectors and adjacency matrix of the graph connection are passed to the FFN blocks which process the nodes and feed them into the GCN layers. The model consists of three GCN layers with activation function ELU using the message passing mechanism. The updated embeddings of the GCN layer are passed to the FFN blocks outputting the final node embedding as logit value. This logit value is then converted into a probability using the Softmax layer, which maps the output to a value between 0 and 1. The model has a combination of ConvLSTM1D with [64, 64] hidden units. The dropout rate is set to 0.3, the learning rate is 0.01, the optimizer Nadam and the batch size 128. The model is trained for 100 epochs with the loss function Sparse Categorical Cross entropy. These configurations are determined based on the ablation study and Bayesian optimization. The result of ablation study and Bayesian optimization, done for the proposed GNN model’s optimization is shown in the “Ablation study” and “Bayesian optimization” section, can be found in “Experiments and results”. For the implementation of the proposed model, we used Intel Core i3-1005G1 processor, 8 GB of memory, and 256 DDR4 SSD storage.

### HOG feature extraction

HOG is a shape descriptor which is widely used for shape feature analysis (Ranjbarzadeh et al. 2022; Cruz-Ramos et al. 2023). The shape pattern characteristics are calculated based on the gradient and edge pixel’s orientation. In terms of breast tumor's shape feature analysis, the HOG is particularly valuable as it can emphasize the mass structures (Sajid et al. 2023). HOG divides the images into smaller portion and calculates the gradient along with its orientation for each section. Then, for each region of the image, a histogram is generated based on the gradients and their orientations. The entire process is known as Histogram of Oriented Gradients, which is a powerful technique for object detection and shape analysis in computer vision. To extract the shape features from raw breast ultrasound images, we have passed 224 × 224 sized image into the HOG feature descriptor. Then, the HOG divides the image into several smaller portion where each portion contains 16 × 16 cells and each cells contain 2 × 2 blocks. After executing the HOG feature descriptor, a feature table is generated with dimensions of 647 × 6084. A graph dataset is generated utilizing the same process shown in “Graph construction” section and fed into the optimized GNN model. The result of this experiment is shown in “Performance comparison between proposed features and HOG descriptor features”, can be found in “Experiments and results” section.

### Experiments and results

The performance and robustness of the proposed GNN model is assessed through several methods including evaluation of performance matrices and statistical analysis. A confusion matrix is generated to calculate the performance matrices. The loss and accuracy curves are shown and k-fold cross validation is conducted to confirm the absence of overfitting issues and performance consistency of the model. In addition, the robustness of the proposed model is assessed using a clustering-based thresholding approach.

### Ablation study

To enhance the performance of the GNN model, an ablation study is conducted changing the hyperparameters. The ablation study comprises nine experiments and for each experiment, the optimal hyperparameter is selected based on the highest accuracy. Table 5 shows the results of nine experiments done for the hyperparameter tuning.

**Table 5:** The results of ablation study

Hidden units			
No.	Hidden units	Test accuracy (%)	Average time per step (s)
**1**	**64,64**	**95.83**	**49**
2	32,32	94.79	45

Learning rates			
No.	Learning rate	Test accuracy (%)	Average time per step (s)
1	0.0001	95.83	49
2	0.001	94.24	49
**3**	**0.01**	**96.35**	**49**

Batch size			
No.	Batch size	Test accuracy (%)	Average time per step (s)
1	32	93.75	91
2	64	95.29	67
**3**	**128**	**96.35**	**49**

Dropout rate			
No.	Dropout	Test accuracy (%)	Average time per step (s)
**1**	**0.3**	**96.86**	**33**
2	0.5	96.34	34
3	0.7	95.29	49

Activation function			
No.	Activation function	Test accuracy (%)	Average tme per step (s)
**1**	**ELU**	**96.86**	**33**
2	ReLU	95.29	33
3	Tanh	92.67	33

Optimizer			
No.	Optimizer	Test accuracy (%)	Average time per step (s)
1	Adam	96.86	33
2	Adamax	95.81	33
**3**	**Nadam**	**97.38**	**33**
4	SGD	92.71	33

Combination type			
No.	Combination type	Test accuracy (%)	Average time per step (s)
1	concat	97.38	33
**2**	**ConvLSTM1D**	**98.44**	**33**

Convolutional layer			
No.	No. of layers	Test accuracy (%)	Average time per step (s)
1	2	98.44	25
**2**	**3**	**99.48**	**33**
3	4	98.57	52

Epoch number			
No.	No. of epochs	Test accuracy (%)	Average time per step (s)
1	50	91.10	26
**2**	**100**	**99.48**	**33**
3	200	99.48	57

The bold column values of each subsection indicate the highest test accuracy achieved within that specific subsection

The first experiment of Table 5 is done altering the hidden units, where with the hidden unit size of [64, 64], the highest accuracy of 95.83% is achieved in 49 s (s). Then, for the learning rate 0.01 and batch size 128, the model performs higher with an accuracy of 96.35%. The two subsequent experiments are focused on altering the dropout rates and activation functions, yielding an accuracy of 96.86% for dropout rate 0.3, and activation function ELU. A reduced time complexity of 33s is obtained with the dropout layer of 0.3 and the complexity remains same for the activation functions. The optimizer Nadam improves the accuracy from 96.86 to 97.38%. The ConvLSTM1D combination performs better than the combination type concat and the accuracy improves to 98.44%. The highest accuracy of 99.48% is obtained for three convolutional layers. In the whole process, the epoch number was set to 100. The last experiment is done altering the number of epochs to 50, 100 and 200. It is observed that, for epoch 50 the performance is not substantial, for 100 and 200 the accuracy remains same at 99.48%, but the time complexity is almost double when the epoch is 200. So, the optimal hyperparameters for the proposed model are: [64, 64] hidden layer, 0.01 learning rate, batch size of 128, dropout rate 0.3, activation function ELU, optimizer Nadam, combination type ConvLSTM1D, three convolutional layers and 100 epochs and the time complexity for the model’s execution is 33 s.

### Bayesian optimization for hyperparameter tuning

Bayesian Optimization is considered to be a powerful algorithm for automating the fine tuning of hyperparameters for complex machine learning, deep learning and other models that require optimization. It is a prominent approach for enhancing the performance of black-box functions that are expensive to evaluate (Dhillon et al. 2023). In the ablation study, we have systematically modified the hyperparameters for gaining the model’s optimal performance. In this section, an automatic approach for tuning the quantitative hyperparameters such as hidden layer, learning rate, batch size, and dropout rate is introduced. Table 6 shows the range of hyperparameters used in the experiment of Bayesian Optimization.

**Table 6:** Hyperparameters setting

Hyperparameter	Range
Hidden layer	32–64
Learning rate	0.001–0.01
Batch size	64–128
Dropout rate	0.3–0.7

The Bayesian Optimization is implemented employing the Gaussian Process model as a surrogate model. The model employs an Upper Confidence Bound (UCB) acquisition function to select evaluation points and balance between exploration and exploitation while searching for promising parameter values (Dhillon, et al. 2023). In this experiment, Bayesian optimization yields optimal hyperparameters: a batch size of 128, a dropout rate of 0.3, hidden units of approximately 64, and a learning rate of 0.01. These hyperparameters led to a remarkable validation accuracy of approximately 99.5%. The automatically selected hyperparameters matches with the hyperparameters obtained from the ablation study which validates the effectiveness of this intelligent optimization technique in enhancing the model’s performance.

### GNN model’s performance analysis

The relational edge table comprises a large number of connections (edges) between the nodes. The presence of such numerous edges can introduce noise, redundancy, and potentially irrelevant information and a higher complexity (Abadal et al. 2022) into the model. An experiment is conducted with several correlation threshold values of node connections to decrease the number of edges and enhance the model’s robustness. In order to find the optimal output with the minimal number of edges, an experiment is carried out which is detailed in Table 7.

**Table 7:** Performance with different threshold values

SI. No.	Threshold	Number of edges	GNN accuracy (%)
1	Nan	208,981	95.83
2	≥ 0.7	154,593	96.35
3	≥ 0.8	113,272	99.07
4	≥ 0.9	49,167	98.96
5	≥ 0.95	30,310	99.48
6	≥ 0.99	4740	97.92
7	= 1.0	1136	97.40

Table 7 lists the threshold for the Spearman correlation score and the number of edges for a specific threshold where “Nan” means no threshold is considered. It can be observed that with increasing the threshold values, the number of edges decreases and the performance improves. For large threshold values, a high correlation between the nodes exists which improves the accuracy. While utilizing all the edges (208,981), a test accuracy of 95.83% is achieved. The highest test accuracy of 99.48% is obtained for a threshold value of 0.95 which means that only the edges for which the Spearman correlation score is equal or greater than 0.95 are considered. This results in an optimized model and reduces the number of edges significantly, from 208,981 to 30,310.

### Performance analysis of the optimized GNN model

**Fig. 11:**
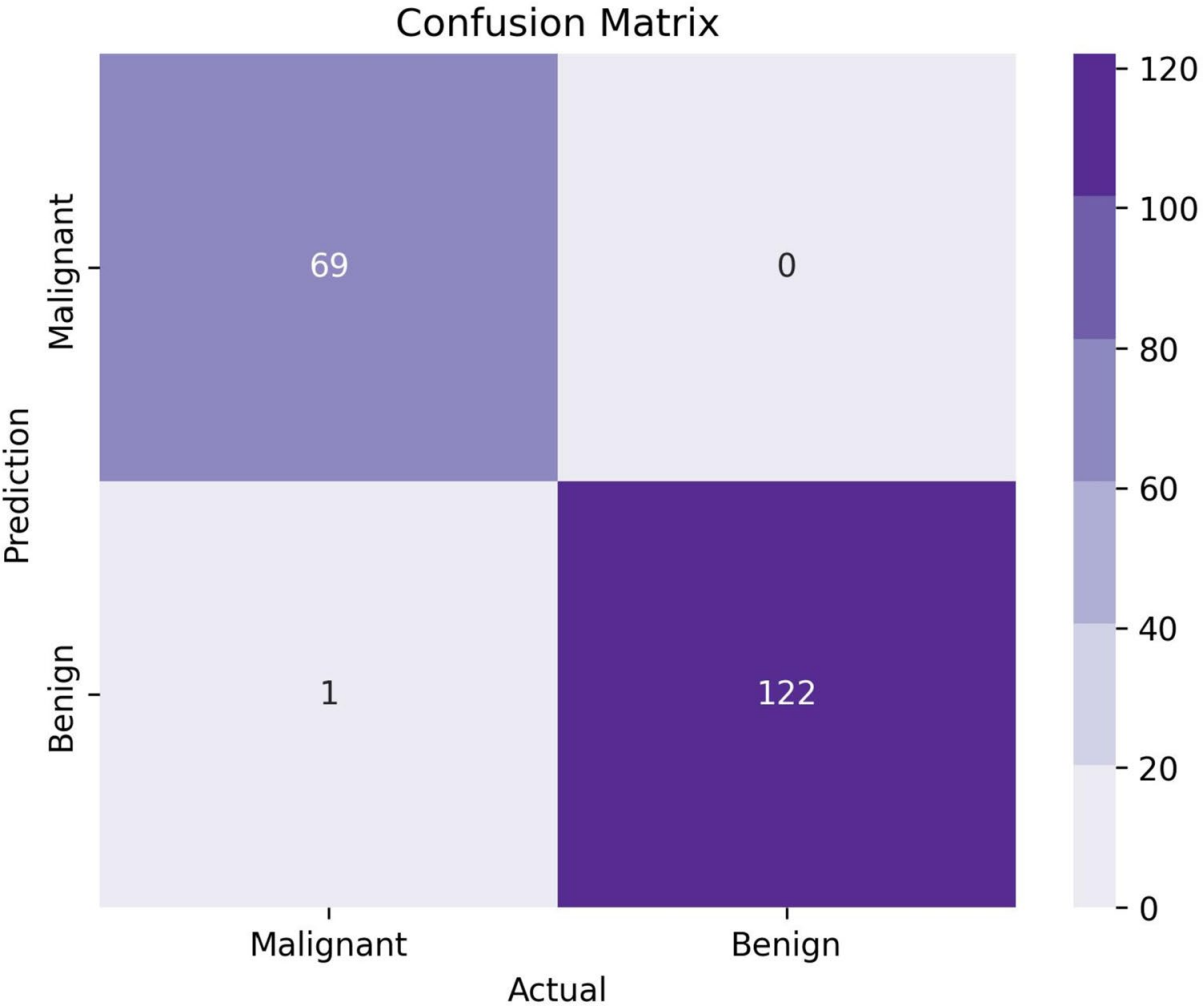
Confusion matrix of the GNN model

The optimized model performs with the highest test accuracy of 99.48% with 30,310 edges. To further analyze the performance, several performance metrics are derived from the confusion matrix of the proposed model, see Fig. 11.

The column values of the confusion matrix in Fig. 11 represent the predicted labels while the rows represent the actual labels. The diagonal values of the confusion matrix indicate the number of accurately predicted test data (Khan et al. 2022). Our findings demonstrate that the model does not exhibit superior predictions for any particular class over others. The evaluation incorporates a diverse range of metrics, including Precision, Recall, F1 score, Specificity, Sensitivity, Negative Predictive Value (NPV), False Positive Rate (FPR), False Discovery Rate (FDR), False Negative Rate (FNR), Matthews Correlation Coefficient (MCC), and overall Accuracy. The results are calculated using the true positive, true negative, false positive and false negative scores of confusion matrix. Table 8 represents the results.

**Table 8:** Performance evaluation of the proposed GNN model

Performance metrics	Results (%)	Performance metrics	Results (%)
Train accuracy	98.55	NPV	99.19
Test accuracy	99.48	FPR	0.00
Validation accuracy	99.78	FDR	0.00
Sensitivity	98.57	FNR	1.4
Precision	100	F1 Score	99.28
Specificity	100	MCC	98.88
Recall	100		

**Fig. 12:**
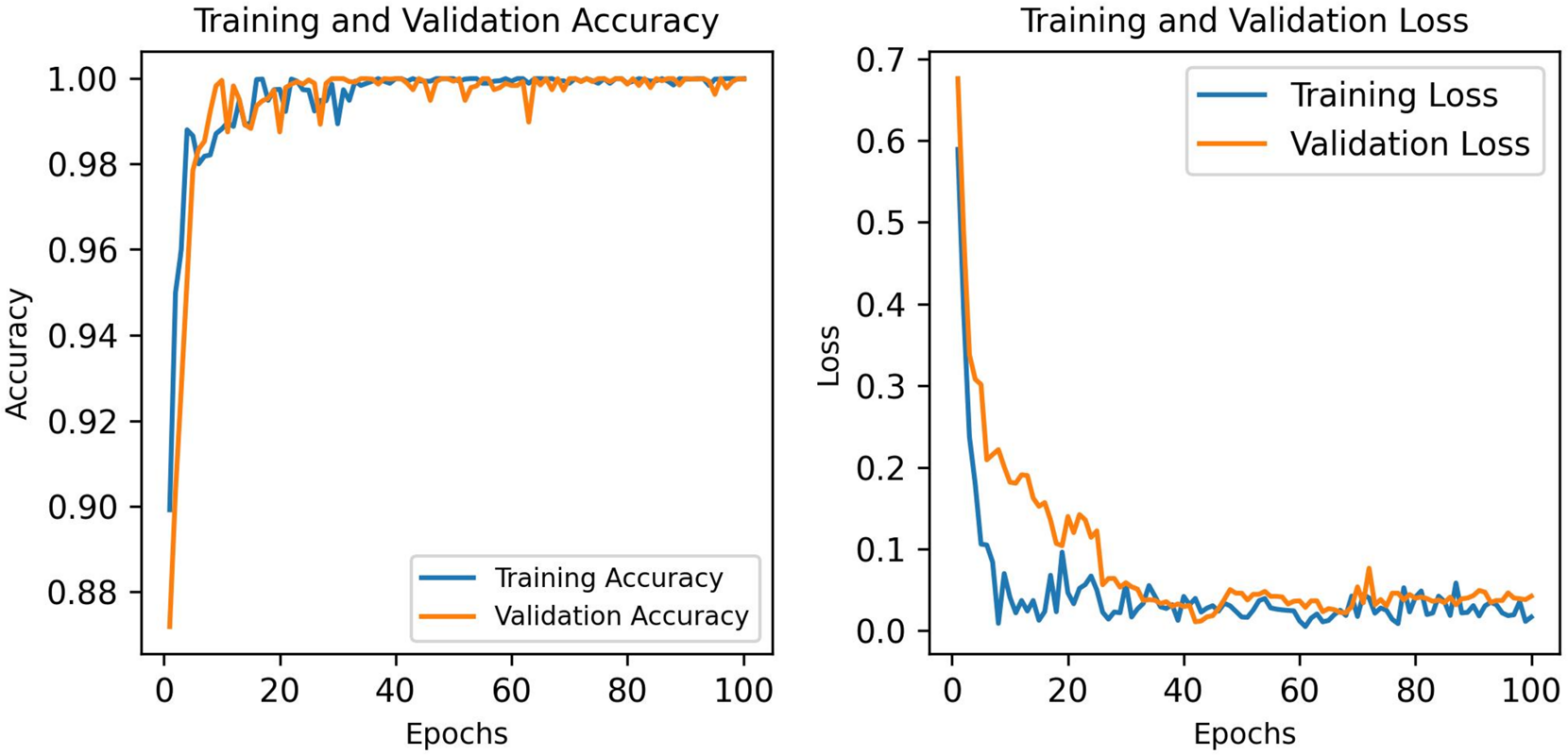
The loss curve and accuracy curve for the proposed GNN model

It can be seen from Table 8 that along with a test accuracy of 99.48%, the model achieves an F1 score of 98.28%, while precision, recall and specificity are all 100%. The train, validation and test accuracies are very close, showing no signs of overfitting. The sensitivity of the model is 98.57% and both FPR and FDR records a score of 0%. The NPV score for the proposed model is 99.19% and the FNR and MCC are 1.4%, and 98.88% respectively. The model’s loss and accuracy curves are shown in Fig. 12.

The loss curve and accuracy curve for each epoch exhibit a consistent pattern, with no significant variation. This validates the model’s robustness (Montaha et al. 2021), indicating no occurrence of overfitting.

### K-fold cross-validation

**Fig. 13:**
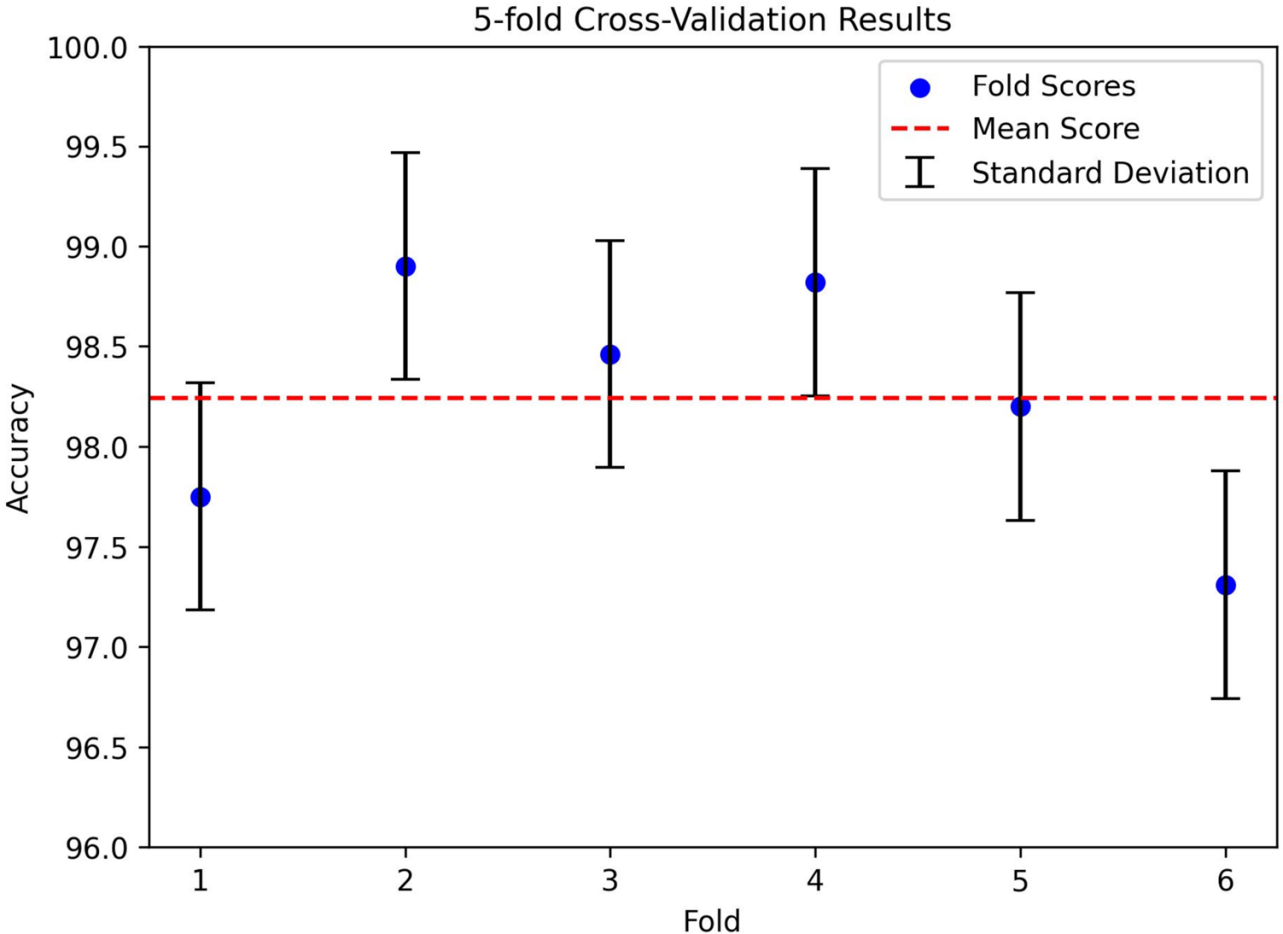
K-fold cross validation on the GNN model

The performance of the proposed model is evaluated using a k-fold cross-validation strategy with fivefolds, where the dataset is separated into five distinct subsets for training and evaluation purposes. The scatter plot in Fig. 13 illustrates the fold-wise accuracy scores, with error bars representing the standard deviation. The red dashed line represents the mean accuracy of 98.05%. The accuracy scores obtained for the individual folds are 97.75%, 98.90%, 98.46%, 98.82%, and 98.20% respectively, reflecting a consistent performance of the model across each subset. The mean accuracy across all folds is 98.05%. These findings highlight the robustness and consistent performance of the proposed GNN model.

### Performance comparison with prior studies

Table 9 provides a comparative overview of prior studies aligned with our proposed methodology. These studies are done employing the same dataset as we have used. A comparison is carried out based on the test accuracy achieved in the classification of breast cancer using the BUSI dataset. The following table includes the information of authors, dataset, model, and result.

**Table 9:** Comparison with previous literatures

Authors	Dataset	Model	Result (test accuracy)
Deb and Jha (2023)	BUSI	Fuzzy rank-based ensemble learner based on four base learners: VGG-Net, DenseNet, Inception Net, and Xception Network	85.23%
Zhuang et al. (2021)	1. BUSI	Bilateral filtering for image processing, VGG19 model for deep feature extraction, Adaptive spatial feature fusion for classification	95.48%
	2. OMI		
	3. Dataset B		
	4. Hospital		
Moon et al. (2020)	1. Seoul National University Hospital (SNUH) dataset	VGGNet, ResNet, and DenseNet	94.62%
	2. BUSI		
Byra (2021)	BUSI	A transfer learning technique based on deep representation scaling (DRS) layers	91.5%
Mishra et al. (2021)	BUSI	Random forest classifier, gradient boosting classifier, AdaBoost classifier (ABC), support vector machine classifier, decision tree classifier, and logistic regression	97.4% (for ABC)
Umer et al. (2022)	1. BUSI	A multiscale CNN classification model comprising 21 layers for classification, an autoencoder-based u-shaped DDA-Net segmentation model	97.89%
	2. UDIAT		
Özcan (2023)	BUSI	BUS − CAD. Hybrid feature representation with global and local texture statistics, feature selection. Highest classification accuracy with random forest (RF) classifier	97.81%
Sirjani et al. (2023)	1. BUSI	Deep neural network architecture based on Inception-V3	81%
	2. BUS		
	3. A public dataset of 86 breast cancer ultrasound images		
Our proposed model	BUSI	Feature extraction, feature selection, graph generation, and classification with GNN	99.48%

From the Table 9, it is observed that, most of the studies are done based on the dataset BUSI, resulting accuracies of 81% to 98% approximately (Zhuang et al. 2021; Moon et al. 2020; Sirjani et al. 2023) introduced deep learning and transfer learning-based classifiers for multiple datasets, resulting an accuracy of 95.48%, 94.62% and 81% respectively. In addition, the time complexity for these models is much higher than our proposed model. (Özcan 2023) introduced a BUS−CAD system that includes global and textural-based feature statistics, feature selection and machine learning classification, achieved an accuracy of 97.81%. When compared with the test accuracies of the prior studies, it is evident that our proposed model has obtained the highest accuracy of 99.48%. The overall comparison with prior studies validates the efficiency of our model in predicting breast cancer at lower time complexity with higher classification accuracy.

### Performance comparison between proposed features and HOG descriptor features

In this section, the GNN model’s performance based on the ten features extracted from breast ROI is compared with the performance of GNN for the HOG descriptor features. The objective of this experiment is to assess the performance of the proposed GNN model using raw ultrasound images, without any prior selection of tumor regions. Table 10 shows the performance comparison.

**Table 10:** GNN model’s performance comparison with handcrafted features and HOG descriptor features

GNN performance on features	Accuracy (%)	Sensitivity (%)	Specificity (%)	Precision (%)	F1 score (%)
Ten handcrafted features	99.48 (Threshold ≥ 0.95)	98.57	100	100	99.28
	95.83 (No threshold)	95.71	95.90	93.06	94.37
HOG descriptor features	86.91 (No threshold)	83.33	88.8	79.71	81.48

Table 10 demonstrates a significant performance difference between the two processes. The GNN model’s performance for HOG descriptor features is approximately 12.57% lower. But the best performance of the proposed GNN model is gained considering strong relationships (threshold ≥ 0.95) between the images based on their feature values. Considering no threshold values for the relationships between nodes, the GNN model obtains 95.83% with the medically significant handcrafted features which is still 8.92% higher than the HOG descriptor features. The performance of sensitivity, specificity, precision and f1 score for the GNN classification is also remarkably lower with HOG descriptor features compared to the other two methods. The noteworthy performance difference is occurred for several reasons. Firstly, the raw breast ultrasound images contain high amount of speckle noise which results poor visualization and tumor tissue boundary minimization (Ayana et al. 2022). Therefore, the raw image may contain redundant features which can mislead the classification model extensively. To address the issue, substantial preprocessing may be required to obtain a noise-free image. However, the introduction of such preprocessing techniques and large number of features for the classification can significantly raise the computational demands constraining overall efficiency. Therefore, the utilization of specific region can reduce unwanted redundant features and noise effectively without requiring any specific preprocessing techniques. In addition, the importance of handcrafted features based on radiologist’s markers in identifying critical areas of breast tumor is remarkable as they focus only the key areas.

Our proposed model not only gives higher performance at lower computational complexity but also does not require any processing of the images. This validates the importance of extracting features from a selective yet significant portion of image that will minimize the presence of noisy and redundant regions. In term of breast tumor classification task, it is evident from the Table 10 that, a relational graph, generated based on selective features of ROIs outperforms the concept of automatic feature extraction by HOG descriptor from full ultrasound image without selecting any ROI. Moreover, this study ensures the most relevant feature through feature selection which boosts the classification more effectively by mitigating the risk of irrelevant feature application and model’s overfitting.

### Robustness analysis of the proposed model

Usually, a graph with optimal connections among the nodes results in a high accuracy. In our generated graph, it has been observed that certain nodes belonging to distinct classes (benign to malignant) exhibit a strong correlation. Likewise, certain nodes belonging to the same classes (benign to benign or malignant to malignant) exhibit weak correlation. This might have an adverse effect on the performance of the model (Jabeen et al. 2022).

### Clustering based on the correlation between the nodes

Anomaly relationships among the nodes, such as (i) a strong correlation among the nodes of distinct classes and (ii) a weak relation among the nodes of the same classes might hamper the model performance. The entire graph is first clustered based on these two possible occurrences. Table 11 presents the clustering range for the graph.

**Table 11:** Clustering based on the correlations between source and target nodes

Correlation range	Relationship	Cluster	Number of connections in each cluster
< 0.4	Weak	1	2075
≥ 0.7	Strong	2	154,593

Correlations below 0.4 (cluster 1) indicate weak relationships between the target and source columns. 2075 connections between the nodes are found in this range. Similarly, correlations greater or equal to 0.7 (cluster 2) represent a strong relationship between the target and source columns, and 154,593 connections between the nodes are found in this range. The edges of the graphs are filtered, eliminating strong correlations among distinct classes and weak correlations among the same classes.

**Fig. 14:**
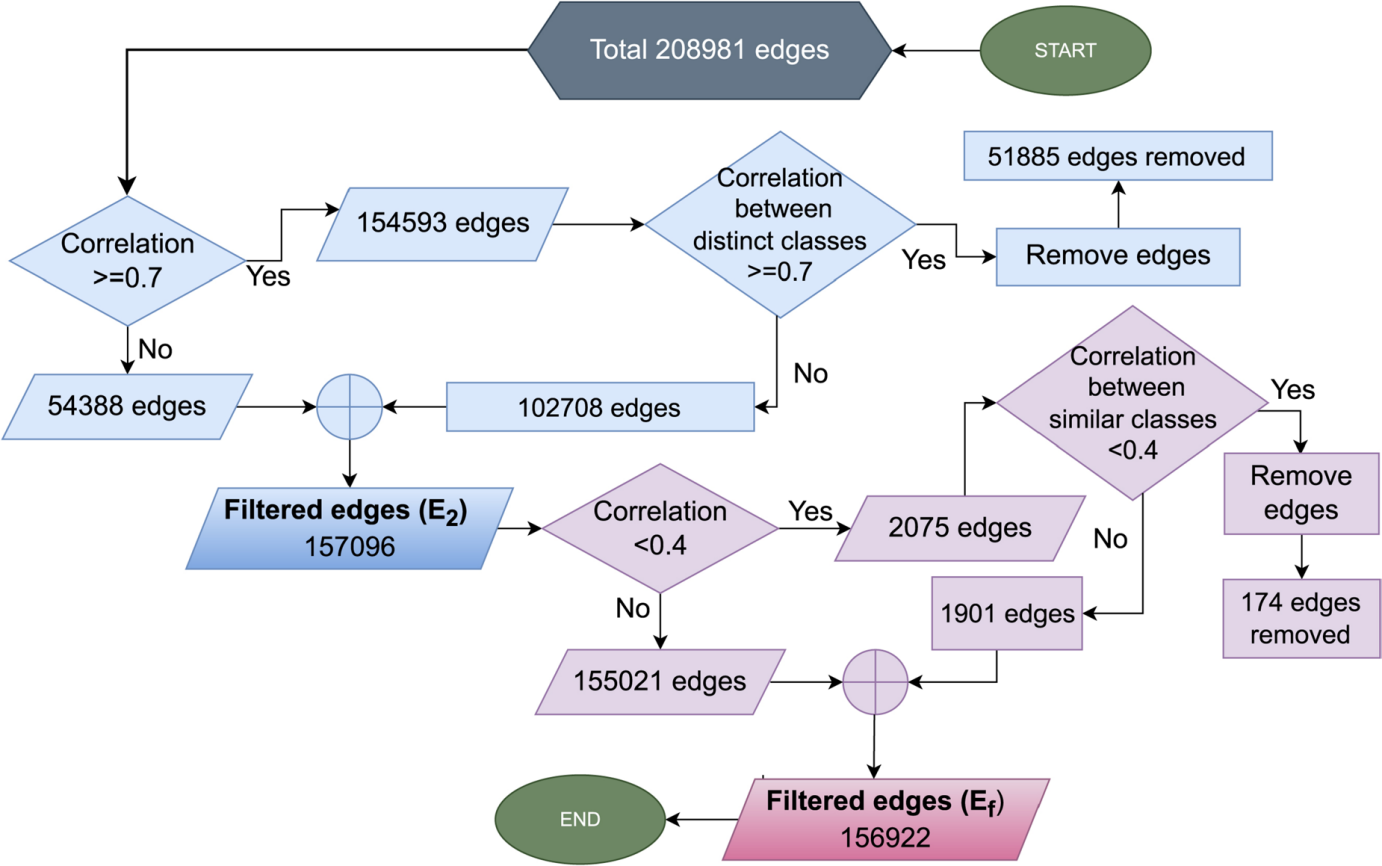
Flow chart of filtering process of the edges

In this filtering process, first the strong relationships among the distinct classes are eliminated. With the resultant edges, we move to the second process of removing weak relationships among the same classes. After completion of the filtering process, a graph is created without strong relationships between different classes and weak relationships between similar classes. This results a filtered graph without anomalies for the edges. For a better understanding, a flow chart of the filtering technique is depicted in Fig. 14.

It is found that 154,593 of the 208,981 edges are above the threshold (≥ 0.7). These include both edges between the same and between different classes. 51,885 edges are found for which strong relationship exist for distinct classes and these are removed. The remaining strong edges are added to the edges with values below the threshold (≥ 0.7), resulting in a total of 157,096 edges. The next step is to check which of these edges have a weak relationship (< 0.4). It is found that 2075 edges are below this threshold. Of these, 174 edges represent a weak relationship for the same classes. These are removed. The final number of edges (*E*_f_) is calculated by adding the 1901 remaining weak edges to the 155,021 edges above the threshold of 0.4. In these final 156,922 edges, there are no edges with strong connections among distinct classes or weak connections among the same classes. Thus, a filtered graph (*V*, *E*_f_) is generated with an updated number of edges.

### Performance comparison of the GNN model with filtered graphs

**Fig. 15:**
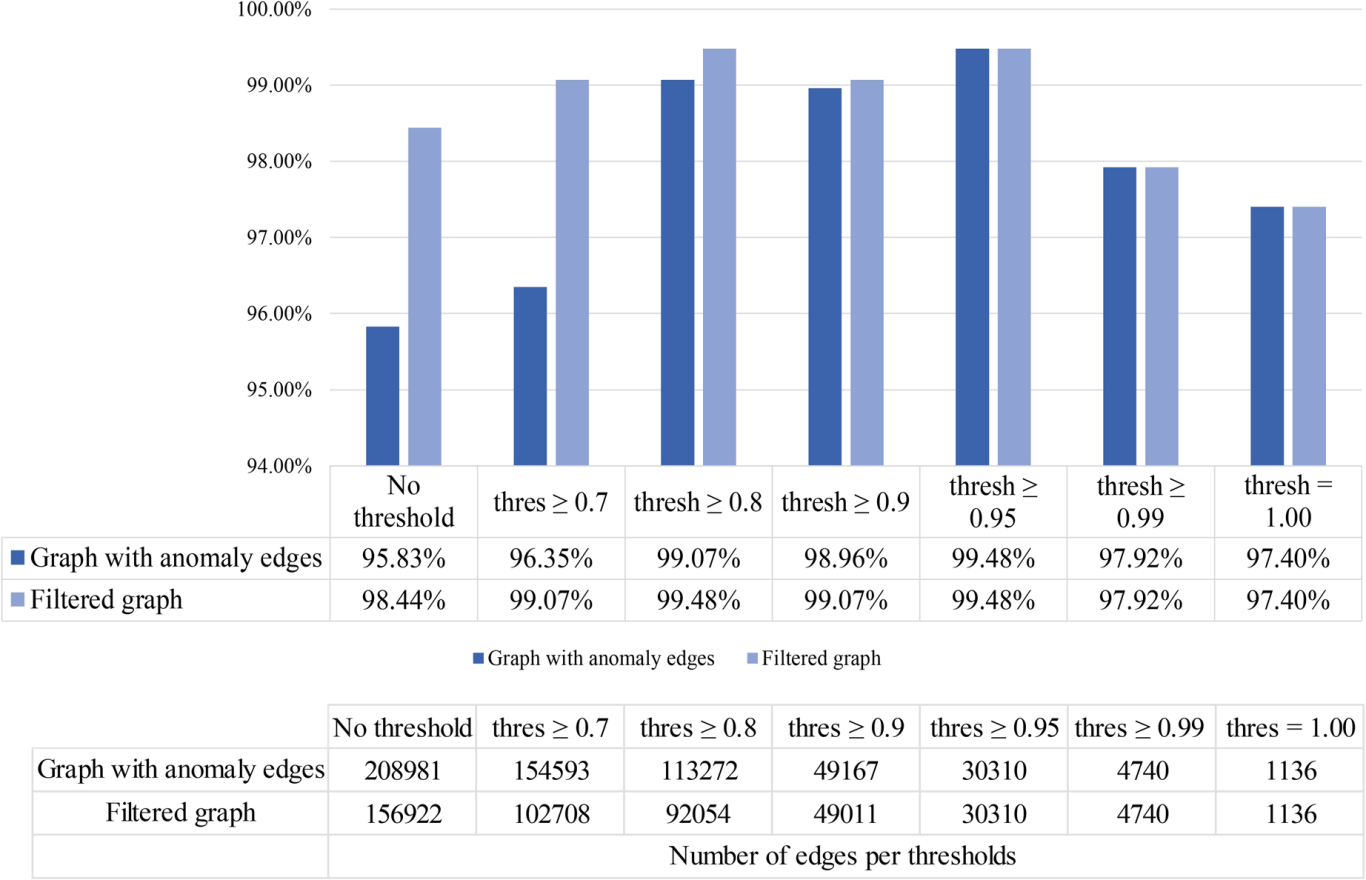
Performance for graph datasets with different thresholds

The filtered graph has a total number of 156,922 edges. We now have two graphs: (i) the first graph before filtering (with anomalies in the edges) and (ii) the second graph after filtering (without anomalies). The proposed model is trained with both graphs and six threshold values for performance comparison. Figure 15 depicts the outcomes.

It can be observed from Fig. 15 that in all the cases, a test accuracy above 97% is achieved for the filtered graph with a highest accuracy of 99.48% for a threshold value of 0.95. Using the first graph with edge anomalies, test accuracy above 95% is achieved across all cases where the highest accuracy, of 99.48%, is acquired while using a threshold value of 0.95. For this threshold, the accuracy and the number of edges are the same for both cases since no edge anomalies are observed at the higher thresholds ($\ge 0.95$) that need to be eliminated for the graph filtering, indicating the effectiveness of the features in distinguishing the classes at higher correlation thresholds.

Compared to the initial graph, an accuracy improved by 2.61% is achieved with the filtered graph when no threshold is considered. While using a threshold of 0.7, the difference in accuracy is approximately 3%. The filtered graph does not contain the 51,885 edge anomalies. At a threshold of 0.8, an improvement in accuracy of 0.41% is observed for the filtered graph. With a threshold of 0.9, the accuracy for the filtered graph increases merely by 0.11%. For both 0.8 and 0.9 thresholds, in the filtered graph, a substantial amount of anomaly edges are removed. For both of the graphs, the test accuracies are very close for these two thresholds, validating that the proposed model is robust enough even with edge anomalies.

### Limitations

Even though the study has achieved a promising result with a GNN-based approach, together with feature extraction and manual graph filtering to classify breast cancer, there are still a certain number of concerns which can be improved in further studies. First of all, the BUSI dataset used in this study is quite small and may not encapsulate the broader amount of characteristic a tumor holds in ultrasonic imaging. The model’s robustness may be enhanced more with a larger dataset that provides diverse patient breast tumor’s ultrasound reports. The performance of the model can be assessed further with a real-world dataset. By doing so, the compatibility of the proposed study can be learnt better as the real-world images contain several challenges. Using other dataset, some other classes of breast cancer can be explored as well. In addition, we aim to analyze the progression of breast cancer in future researches through automated approach. Despite of these limitations, the proposed approach has demonstrated its potential for GNN-based breast cancer classification, offering an effective foundation for future research that aims for optimizing diagnostic accuracy and clinical utility.

## Conclusion

The diagnosis of breast cancer is a major healthcare concern that requires accurate and efficient methods to ensure early detection and proper treatment. This study proposes a novel automated approach for classifying breast cancer into as either benign and or malignant using ultrasound images. Ten informative handcrafted features are extracted from the ROI of breast ultrasound images and a GNN model optimized employing ablation study and Bayesian Optimization, is utilized. The significance of these features is assessed by two statistical analysis methods: density plot and T test. The outcomes demonstrate that the features are suitable to distinguish benign and malignant breast tumors. The GNN model is trained with a graph generated from the features where each image is denoted as node and relationship among the nodes is based on the Spearman correlation coefficient. Several thresholds for the Spearman correlation score are tried with the model and the highest test accuracy of 99.48% is achieved for a threshold value of 0.95 with 30,310 edges. By comparing the model's performance with HOG descriptor features extracted from the full ultrasound image and the handcrafted features extracted from tumor ROI, the significance of ROIs and medically relevant features for breast tumor classification is assessed. The robustness of the proposed approach is further validated with a clustering analysis where weak relationships between similar classes and strong relationships between dissimilar classes are eliminated. The performance of the model with the previous main graph and the filtered graph, without edge anomalies, is compared. For high threshold values, no significant difference is observed. This validates the robustness of the model and the feature set, as the model can achieve optimal performance even with the presence of edge anomalies. The study demonstrates that a graph generated with a set of robust features can play a crucial role in the classification with GNN. The proposed approach might aid the radiologists to diagnose tumors and learn more about tumor pattern based on numerical features.

## Data Availability

The dataset is publicly available and can be accesses through this link: https://scholar.cu.edu.eg/?q=afahmy/pages/dataset.
